# Cannabidiol Prevents Heart Failure Dysfunction and Remodeling Through Preservation of Mitochondrial Function and Calcium Handling

**DOI:** 10.1016/j.jacbts.2024.12.009

**Published:** 2025-02-19

**Authors:** Gerardo García-Rivas, Omar Lozano, Judith Bernal-Ramírez, Christian Silva-Platas, Felipe Salazar-Ramírez, Abraham Méndez-Fernández, Carolina Morales-Ochoa, Hugo Alves-Figueiredo, Martín Rogelio Ramos-González, Nestor Rubio-Infante, Eduardo Vázquez-Garza, Luis A. Luévano-Martínez, Silvia López-Morán, Héctor Chapoy-Villanueva, James Bolton, José-Luis Velasco-Bolom, Paola Mendoza-Espinosa, Flavio F. Contreras-Torres, Carlos Jerjes-Sánchez, Guillermo Torre-Amione

**Affiliations:** a Tecnologico de Monterrey, Escuela de Medicina y Ciencias de la Salud, Cátedra de Cardiología y Medicina Vascular, Monterrey, Mexico; b Tecnologico de Monterrey, Institute for Obesity Research, Monterrey, Mexico; c Cardiol Therapeutics Inc, Ontario, Canada; d Tecnologico de Monterrey, Escuela de Ingeniería y Ciencias, Monterrey, Mexico; e The Methodist Hospital, Cornell University, Houston, Texas, USA

**Keywords:** Ca^2+^ dynamics, cannabidiol, heart failure, mitochondrial energetics, oxidative stress, PPAR-γ

## Abstract

Heart failure (HF) is characterized by energy deprivation, calcium (Ca^2+^) handling alterations, and inflammation: effects associated with mitochondrial dysfunction. Cannabidiol previously prevented mitochondrial dysfunction. Thus, it may prevent HF progression. In mice with HF, subcutaneous cannabidiol attenuated cardiac fibrosis, hypertrophy, loss of ejection fraction, and inflammation; isolated cardiomyocytes preserved cell shortening, Ca^2+^ handling, mitochondrial function and redox balance. Hypertrophied ventricular cardiomyoblasts suggested cannabidiol-mediated effects through peroxisome proliferator-activated gamma receptors. Therefore, cannabidiol in HF limited cardiac hypertrophy and preserved contractile function by sustaining cardiomyocyte and mitochondrial function through redox balance maintenance, supporting cannabidiol role as a cardioprotective therapy in HF.

## Highlights

• The underlying mechanisms of cannabidiol cardioprotection have not been completely determined.
• Subcutaneous administration of cannabidiol in a mouse model of HF resulted in attenuation of cardiac fibrosis and hypertrophy, and improved ejection fraction and cardiac output.
• Cannabidiol-treated HF ventricular myocytes preserved cell shortening and sarcoplasmic reticulum Ca^2+^ uptake concomitant with the maintenance of mitochondrial function and redox balance.
• Cannabidiol-treated angiotensin II-induced hypertrophic ventricular cardiac myoblasts suggest a PPAR-γ–dependent mechanism for its cardioprotective effect.

## Visual Abstract

**Figure:**
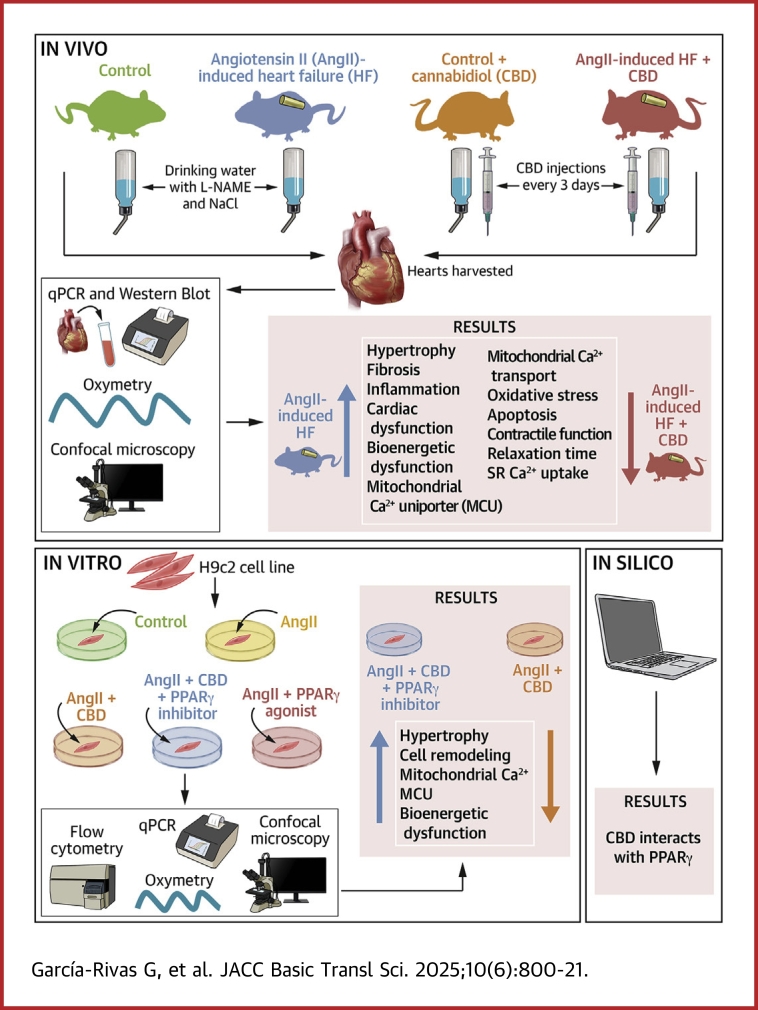

Heart failure (HF) progression is characterized by progressive fibrosis, inflammation, decreased contractile function and relaxation, as well as apoptosis. Central to the underlying mechanisms that are responsible for all those changes is the state of mitochondrial function. In failing myocardium alterations in Ca^2+^ handling, respiratory chain and electron transport and ultimately energy supply are seen, and therefore, preserving mitochondrial function provides a unique therapeutic target.

Contractile dysfunction and pathological remodeling associated with HF are closely related to Ca^2+^ mishandling;^1^^,^^2^ Ca^2+^ is a second messenger that links electrical stimulation of the plasma membrane with the cytosolic Ca^2+^ handling and ATP production in mitochondrial excitation- contraction-energetic coupling.^3^^,^^4^ The major pathway for mitochondrial Ca^2+^ uptake is the mitochondrial calcium uniporter (MCU) complex, which is overexpressed in murine models of pathological hypertrophy^5^, ^6^, ^7^ and in the left ventricle (LV) of patients with HF, positively correlating with pathological remodeling.^8^ In this context, cannabidiol has been shown to be beneficial, because it directly targets the mitochondria and modulates mitochondrial Ca^2+^ handling in several diseases, including cardiac disorders.^9^, ^10^, ^11^, ^12^

Cannabidiol is a major constituent of the plants of the genus *Cannabis* and, in recent years, evidence has accumulated in support of a cardioprotective effect mediated by cannabidiol, including limiting damage caused by ischemia-reperfusion injury.^13^, ^14^, ^15^, ^16^ Cannabidiol can counterbalance chemotherapy cardiotoxicity by attenuating oxidative stress, boosting mitochondrial function, favoring mitochondrial biogenesis, reducing inflammation, diminishing cell death, and safeguarding myocardium ultrastructure.^17^^,^^18^ From a chronic treatment perspective, cannabidiol was shown to partially rescue the heart from functional and molecular alterations related to diabetic cardiomyopathy, presumably by dampening oxidative-nitrosative stress and inhibiting the nuclear factor kappa beta (NF-κβ) pathway.^19^ Moreover, cannabidiol relieved the heart from both systolic and diastolic dysfunction by hampering T-cell infiltration in a preclinical model of autoimmune myocarditis.^20^ Because of its demonstrated safety and tolerability, along with the previously indicated evidence of its cardioprotective action,^21^ cannabidiol has great translatability in the field of cardiovascular disorders. However, the mechanisms by which cannabidiol confers cardioprotection are not completely understood.

In the present study, we hypothesized that cannabidiol prevents progression of HF by preserving the mitochondrial function. In an angiotensin II (ANGII)-induced HF mouse model we evaluated whether cannabidiol improves cardiac remodeling and inflammation. Furthermore, the therapeutic effects of cannabidiol were explored over cardiac function in the HF heart and in isolated HF cardiomyocytes. In addition, cardiac bioenergetics, mitochondrial Ca^2+^ overload, and oxidative state were assessed in HF cardiomyocytes and an in vitro model of cardiac hypertrophy. Finally, the role of cannabidiol in the peroxisome proliferator–activated receptor γ (PPAR-γ) was determined by an in vitro model of cardiac hypertrophy and in silico studies. The results point towards mitochondrial protection via PPAR-γ activation by cannabidiol (CBD).

## Methods

### Reagents

All reagents were purchased from Sigma-Aldrich, unless otherwise stated. Cannabidiol was obtained from Noramco.

### Experiments performed: in vivo, ex vivo, and in vitro

A summary of the performed experiments in this study can be found in Supplemental Figure 1. In vivo studies were complemented with ex vivo and in vitro studies to characterize the mechanisms of action of cannabidiol on cardiac tissue.

### HF animal model

All animal protocols were reviewed and approved by the Institutional Animal Care and Use Committee of the Tecnológico de Monterrey in accordance with the NORMA Oficial Mexicana NOM-062-ZOO-1999 (Protocol number 2018-003) following the ARRIVE guidelines. Male C57BL/6 mice purchased from Bioinvert. Heart failure was induced in 11-week-old mice after administration of 1% NaCl and 0.01% of N-nitro-L-arginine methyl ester (L-NAME) in the drinking water for 1 week. Then, a micro-osmotic pump was surgically implanted in the subdermal dorsal area diffusing ANGII at a rate of 0.7 mg/kg/d.^22^ The control group received the same manipulation, without the implantation of the pump. Animals were kept at 25 °C with a 12-hour light/dark cycle. Water and food were given ad libitum. A group of ANGII-treated animals were injected subcutaneously with cannabidiol dissolved in polyethylene glycol and elastin-like polypeptide (PEG-ELP) every third day for 28 days, the second day after pump implantation (HF+CBD). The other groups were given an equivalent volume of vehicle. On the 28th day after the pump implantation, the end of the protocol, one group of animals was used for pressure-volume (PV) loop analysis. The hearts of these animals were preserved for protein and RNA extraction, histopathologic analysis, and mitochondrial isolation. Other groups of animals were used to isolate cardiomyocytes.

### Euthanasia and tissue selection

At the end of the protocol, mice were first anesthetized with sevofluorane 4% to 5% before any procedure. In this case, a laparotomy-style incision in the abdomen was performed to obtain at least 500 μL of blood from the vena cava. Afterwards, hearts were excised and placed in a petri dish with phosphate-buffered saline (PBS) solution. Hearts were weighed and cut into 3 sections. Middle sections were placed in a formaldehyde fixating solution, whereas the apex and the rest were placed into liquid nitrogen, then moved to −20 °C storage for polymerase chain reaction (PCR) analysis. In another set of experiments, PV loops were measured along with mitochondria extraction. Here, mice were intubated. Afterwards, the heart was accessed through a laparotomy-style incision in the abdomen and a catheter was inserted into the LV. After PV loop recording, the heart was excised and transferred to a tube with mitochondria isolation solution. In another set of experiments cardiomyocytes were isolated, where the heart was accessed through a laparotomy-style incision in the abdomen to inject cardiomyocyte isolation solution directly into the right ventricle to wash as much blood as possible. Afterwards, the ascending aorta was clamped, the heart was excised and placed into a petri dish with isolation solution to continue the protocol ex vivo, and more isolation solution was injected directly into the LV to pass through coronary circulation.

### Histopathologic slides preparation and microphotography

Samples for histopathologic analysis were fixed in 4% (weight/volume) paraformaldehyde in PBS for at least 24 hours at room temperature, transferred to 70% ethanol, embedded in paraffin, and processed for Masson’s trichrome and hematoxylin and eosin (H & E) staining. Microphotographs were acquired using an Imager Z.1 Zeiss microscope, with an AxioCam HRm and microphotograph processing with the AxioVision software. These were used to assess cardiac fibrosis and myocyte, as detailed in the following text.

### Assessment of cardiac remodeling by fibrotic index and hypertrophy

To assess fibrosis, we used a semiquantitative approach; after staining with Masson's trichrome, we took blinded pictures of the whole tissue and quantified the number of color pixels of each microphotography to make a ratio of %blue (fibrotic tissue)/%red (muscle) using ImageJ software.^23^ Heart hypertrophy was assessed by normalizing heart weight to body weight and by assessing the area of transversal myocytes by taking pictures from H & E–stained slides. A total of 2 to 3 photos were taken for each slide at the level of the papillary muscles. Only cells with the nucleus at the center and with a defined cytoplasm were considered. At least 20 cells per image were selected, and the cross-sectional diameter was assessed with a protocol as previously reported,^24^ using the AxioVision software.

### Terminal deoxynucleotidyltransferase-mediated dUTP-biotin nick end labeling assay

For analysis of apoptosis, we used the HRP-DAB terminal deoxynucleotidyltransferase-mediated dUTP-biotin nick end labeling assay staining kit (Ab206386, Abcam); 10 μmol/L sections of paraffin-embedded hearts were processed according to the manufacturer’s instructions. All of the stained slides were scanned, blinded to the user, with a Imager Z.1 Zeiss with AxioCam HRm and processed using the AxioVision software (Zeiss). The measurement of relative apoptosis in tissues was performed with the pixels count analysis using the ImageJ software.

### Caspase activity measurements

Apoptosis was determined in 100 μg of heart homogenates, as we described previously,^25^ using the Caspase Glo 3/7 assay (Promega) according to manufacturer instructions. The ratio of Caspase-Glo 3/7 Reagent volume to sample volume was 1:1 (in a total of 100 μL), and the maximal luminescent signal was measured after 60 minutes. Luminescence was determined using the Synergy HT plate reader (BioTek). Increased luminescence was assumed to be incremented apoptosis in cardiac cells.

### In vivo intra-LV hemodynamics

The LV hemodynamics were assessed in vivo through PV analysis performed as previously described,^26^ using an open-heart configuration and a 1.2-F PV catheter with the ADV500 PV measurement system (Transonic Science). At least 5 mice were used to measure heart hemodynamics for each group; data were acquired and analyzed using labScribe version 4.31 (iWorx System Inc).

### In vivo blood pressure assessment

For blood pressure assessments, animals were acclimated to a holder for 3 consecutive days before the actual determination of blood pressure during the last week of the model. Acclimation consisted of gently placing each animal for 3, 5, and 10 minutes inside a rodent holder during the first, second, and third training days. All sessions, including training, took place from 9:00 am to 11:00 am. No anesthesia was required. After 3 consecutive days of acclimation, blood pressure was measured noninvasively in the animal’s tail with the CODA system (Kent Scientific). Once inside the holder, the animal was placed over a heating pad so that the tail temperature was about 32 °C. A ring- like “occlusion cuff” was slid to the base of the tail until resistance was encountered. A volume-pressure recording “VPR cuff” was placed behind the occlusion cuff. The animal was then covered with a warming blanket. The initial 5 cycles were discarded, after which 10 regular cycles were recorded. The deflation time of the occlusion cuff was set to 20 seconds. At least 3 “accepted” readings per animal, according to the CODA software (Kent Scientific), were considered for further analysis.

### Cardiomyocyte isolation

The procedure was performed following previous reports.^27^ After anesthesia, in C57/BL6J mice, the chest was opened to expose the heart and the descending aorta was cut. The heart was flushed by injection of EDTA buffer (in mmol/L: 130 NaCl, 5 KCl, 0.5 NaH_2_PO_4_, 10 HEPES, 10 glucose, 10 BDM, 10 taurine, 5 EDTA) into the right ventricle. Ascending aorta was clamped and the heart was transferred to a dish to inject EDTA buffer, perfusion buffer (in mmol/L: 130 NaCl, 5 KCl, 0.5 NaH_2_PO_4_, 10 HEPES, 10 glucose, 10 BDM, 10 taurine, 1 MgCl_2_), and collagenase buffer (in mg/mL: 0.5 Coll 2, 0.5 Coll 4, 0.05 Protease XIV 0.05) into the LV. Constituent chambers were separated using forceps. Cellular dissociation was completed by gentle trituration with a 1-mL syringe, and enzyme activity was inhibited by addition of 5 mL stop buffer (5% fetal bovine serum [FBS] in perfusion buffer). Cell suspension was filtered and the extracellular Ca^2+^ concentration was gradually restored before continuing with the following experiments.

### Mitochondrial membrane potential measurement in intact cardiomyocytes

Isolated cardiomyocytes were incubated in Tyrode (Ty) buffer (in mmol/L: 128 NaCl, 0.4 NaH_2_PO_4_, 6 glucose, 5.4 KCl, 0.5 MgCl, 5 creatinine, 5 taurine, and 25 HEPES; pH 7.4) supplemented with 1 μmol/L Ca^2+^ and 300 nmol/L tetramethylrhodamine ethyl ester perchlorate (T669, Thermo Fisher Scientific) for 30 minutes at 25 °C.^28^ After washing with fluorophore-free and Ca^2+^-free Ty buffer, 2-dimensional images (1,024 × 1,024 pixels, 400 Hz, 1-μm section thickness) were taken at 543 nm excitation and 555 to 700 nm emission window to register the mitochondrial membrane potential (ΔΨm). Results are shown normalized to the control group as a percentage analyzed by imageJ.

### Mitochondria isolation

After the 28th day of treatment, hearts were excised and cut into small pieces. Minced hearts were washed in digestion buffer (in mmol/L: 250 sucrose, 1 EDTA, and 10 Hepes pH 7.3) and incubated in digestion buffer supplemented with 0.12 mg protease (subtilisin A). Samples were incubated for 10 minutes at room temperature. Afterwards, samples were centrifuged at 800 *g* for 10 minutes. Clarified homogenates were centrifuged at 10,000 *g* for 10 minutes to recover cardiomyocytes. The pellet was suspended in mitochondrial isolation buffer (in mmol/L: 250 sucrose and 10 Hepes pH 7.3) and broken in a Dounce homogenizer. Mitochondria were obtained by differential centrifugation according to Bernal-Ramírez et al.^29^

Isolated mitochondria were suspended in mitochondrial respiratory buffer (in mmol/L: 140 potassium gluconate, 5 KH_2_PO_4_, and 10 EPES, pH 7.2) and diluted at a final concentration of 0.6 mg/mL.

### Mitochondrial function assessment

Respiratory studies were performed in isolated mitochondria, which isolated from hearts and diluted in mitochondrial respiratory buffer to obtain a final concentration of 0.1 mg/mL. Respiratory chain activities were evaluated in a high-resolution respirometer (Oroboros Instrument) by measuring the succinate-dependent respiration in the presence of 10 mmol/L succinate and 2 μg/mL rotenone.^28^ Routine and phosphorylating respiration (state 3) were assessed by the sequential addition of substrate (10 mmol/L succinate + 2 μg/mL rotenone), 200 μmol/L ADP, and 0.08 μmol/L carbonyl cyanide p-trifluoro-methoxyphenyl hydrazone (FCCP), respectively.

### Oxidative stress markers

Protein carbonyl content was assessed in homogenized cardiac tissue following the assay kit ab126287 (Abcam) instructions. Absorbance was measured was measured using a Synergy HT plate reader (BioTek).

Reduced glutathione (GSH) and oxidized glutathione (GSSG) were measured in homogenized cardiac tissue following the assay kit ab239709 (Abcam) instructions. Absorbance was measured was measured using a Synergy HT plate reader (BioTek).

### Cell shortening and Ca^2+^ handling measurements in intact cardiomyocytes

Cell shortening and intracellular Ca^2+^ signaling were evaluated simultaneously, as previously reported.^30^ Data analysis was performed in MATLAB version 9.4.0.813654 (R2018a) (The MathWorks, Inc). Transient characteristics evaluated were transient amplitude as the maximal ΔF/F_0_ value, where F_0_ is the average fluorescence intensity before Ca^2+^ transient rise; time to peak as the time between the onset and the peak points; time to 50% of decay (T_50%_) as the time elapsed between the peak and 50% amplitude time points; and decay tau (τ) as the time constant of a simple exponential fit of the decay phase of the transient, comprised between the peak and the ending times. Cell shortening was characterized as maximal shortening as the maximal difference between cell lengths. Time to peak shortening (TTPS) is the time elapsed between the onset and the maximal shortening points; and time to half relaxation (TTHR) is the time elapsed between the maximal shortening time and the moment by which the cell length has recovered to 50% of the maximal shortening. To evaluate spark characteristics in freshly isolated myocytes, longitudinal cell axis with 100-nm pixel size records were taken at a 1 Hz pace. Analysis was performed using ImageJ software (National Institutes of Health) with the Sparkmaster plugin.^31^ All of the confocal measurements were acquired using a Leica TCS SP5 confocal microscope equipped with a D-apochromatic 40X, 1.2 NA, oil objective (Leica Microsystems) at 25 °C.^28^^,^^29^

### Cellular culture

Rat ventricular myocardial H9c2 cell line (CRL-1446, ATCC) passage 20 was seeded in 6-well plates in Dulbecco's Modified Eagle's Medium-high glucose medium (Sigma Aldrich, Cat nr: D- 7777) + 10% FBS and 1% penicillin +streptomycin at 70,000 cells/well for reactive oxygen species (ROS) assessment and 20,000 cells/well for hypertrophy evaluation. Incubation conditions were 37 °C and 5% CO_2_. After 24 hours, cells were starved off FBS to 1%; this concentration was kept for the rest of the experiment. The cells received 1 μmol/L ANGII (TOCRIS, R and D), each 24 hours for 2 days, and the respective groups received 0.001, 0.01, 0.1, and 1 μmol/L cannabidiol.

### In vitro assessment of cellular hypertrophy

To evaluate cellular hypertrophy after ANGII stimulation, H9c2 cells were seeded in coverslips and loaded with calcein-AM (5 μmol/L, Invitrogen, C34852) during 30 minutes at 37 °C. Afterward, coverslips were mounted in a superfusion chamber and Draq5 (20 μmol/L, Thermo Scientific, 62251) in Tyrode was added to stain the nuclei. XY records were taken using a Leica TCS SP5 confocal microscope equipped with a D-apochromatic 40×, 1.2 NA, oil objective (Leica Microsystems), excitation wavelength was 488 nm, and the emission window was 500 to 600 nm. Images were analyzed using ImageJ software (National Institutes of Health) to assess cell surface area.

### In vitro assessment of mitochondrial ROS

H9c2 cells were detached and resuspended in 500 μL of Tyrode with 5 μmol/L MitoSOX incubated for 10 minutes at 37 ^o^C and analyzed by flow cytometry measuring fluorescence. At least 20,000 events were analyzed in a FACSCanto II cytometer (BD Biosciences) and triplicate experiments were carried out for each set. We performed doublet exclusion for each analysis and analyzed the MFI expression of the whole population of MitoSOX (PE channel) and DCFDA (FITC channel). Data were analyzed using FlowJo V10.

### In vitro mitochondrial Ca^2+^ content

H9c2 cells were suspended in respiratory buffer supplemented with 0.04 μmol/L digitonin, 1 nmol/L Fluo-4 AM (Life Technologies). The cell impermeant probe Fluo-4 can sense intracellular Ca^2+^ once cells are permeabilized with digitonin. This allows for the monitoring of cytosolic Ca^2+^ given that the probe is not permeable to mitochondria. Cytosolic Fluo-4 fluorescence (*F*_*0*_ value) was then measured in a Synergy HT microplate reader (filters ex/em: 490/530 nm). After *F*_*0*_ determination, the Ca^2+^ contained within mitochondria was determined by depolarizing mitochondria with the respiratory chain uncoupler FCCP (1 μmol/L). Mitochondrial depolarization allows release of the mitochondrial Ca^2+^ to the cytosol, which is now sensed by Fluo-4 retained in the cytosol, thus obtaining an *F* value corresponding to the Ca^2+^ contained in mitochondria. Free Ca^2+^ signal was calibrated using 100 mmol/L CaCl_2_ and 1 mmol/L EGTA to obtain the *F*_*max*_ and *F*_*min*_ values, respectively. The intramitochondrial free Ca^2+^ content was calculated using the equation:

$$\left[{\text{Ca}}^{2+}\right]=K_{d}\left(F\;-\;F_{\min}\right)/\left(F_{\max}\;-\;F\right)$$

where *K*_*d*_ is the dissociation constant for the Ca^2+^/Fluo-4 couple (K_d_ = 335 nmol/L). Total protein was determined using the Lowry method with the remaining sample (10 μL). All data are finally expressed as Ca^2+^ per mg of sample.

### In vitro mitochondrial Ca^2+^ retention capacity (CRC) and Ca^2+^ influx rate

Mitochondrial CRC was performed as previously described,^29^ which is a measurement used to approximate how much Ca^2+^ can uptake mitochondria relative to healthy cells levels. Briefly, isolated mitochondria were incubated in mitochondrial respiratory buffer supplemented with 0.3 μmol/L Calcium Green-5N (Life Technologies), 10 mmol/L succinate, rotenone (10 μg/mL), 200 μmol/L ADP, and 0.25 μg Oligomycin A for 5 minutes. Afterwards, 10 μmol/L of Ca^2+^ aliquots were added every 3 minutes until the opening of the mitochondrial permeability transition pore (mPTP) as manifested by the massive release of Ca^2+^ from the mitochondrial matrix. This is observed by the increased fluorescence of Ca^2+^ green-5N in the extramitochondrial milieu, and represents the susceptibility of mPTP opening induced by intramitochondrial Ca^2+^. This fluorescence was recorded in a Synergy HT microplate reader at 488 nm excitation and emission of 528 nm.

Additionally, CRC was assessed in permeabilized H9c2 cells. Briefly, 1 × 10^6^ H9c2 cells were harvested and resuspended 2 times in calcium-free Tyrode buffer. Cells were then resuspended in cell respiratory buffer (in mmol/L: 150 sucrose, 5 KCl, 20 Tris-HCl, 2 KH2PO4; pH 7.3) supplemented with 40 μmol/L digitonin, 2 ug/mL rotenone, 12.5 mmol/L succinate, 10 umol/L EGTA, and 1 μmol/L Calcium Green-5N. After stabilization, 10 μmol/L of Ca^2+^ pulses were added, and fluorescence was recorded at 488 nm excitation and 528 nm emission.

To determine Ca^2+^ influx rate in digitonin-permeabilized cells (H9c2 cell line) or cardiomyocytes, 1 × 10^6^ cells were resuspended in cell respiratory buffer supplemented with 0.3 μmol/L Calcium Green-5N (Life Technologies), rotenone (10 μg/ml), and 1 μmol/L cyclosporin A (CSA) to reduce the chance of mPTP opening during the experiment. After fluorescence stabilization, a single 40 μmol/L of Ca^2+^ bolus was added as an extramitochondrial high free-Ca^2+^ reference, then 12.5 mmol/L succinate was added to energize mitochondria, promote a full ΔΨm, and Ca^2+^ influx. Fluorescence was recorded at 488 nm excitation and 528 nm emission in a Synergy HT microplate reader.

Mitochondrial Ca^2+^ influx rate was assessed using the derivative of a second-order polynomial regression adjusted to the fluorescence signal decay.

### RNA extraction and real-time PCR analysis

Total RNA from heart ventricles was extracted using TRIzolReagent (15596026, Invitrogen). Sample purity was evaluated by 260/280 nm absorbance ratio. SensiFAST cDNA Synthesis Kit (BIO-65053, Bioline) was used to reverse-transcribe cDNA from 1 μg of total RNA, and qPCR reaction was performed using SensiFAST SYBR Lo-ROX Kit (BIO-94020, Bioline) in a Quant-Studio 3 RT PCR System (ThermoFisher Scientific). The 2^−ΔΔCt^ method was used to estimate mRNA expression from each gene.^32^ T4 Oligo (Mexico) synthesized primers (Supplemental Table 1).

### Protein purification and Western blot analysis

Cardiac tissue was lysed in radioimmunoprecipitation assay buffer, which contained 50 mmol/L Tris (pH 7.5), 5 mmol/L EDTA, 150 mmol/L NaCl, 1% Triton X-100, 0.1% sodium dodecyl sulfate, 10 mmol/L sodium fluoride, and 0.5% sodium deoxycholate. Phosphatase and protease inhibitor cocktails (Roche) were supplemented to prevent protein degradation. The lysates were subjected to 3 freeze-thaw cycles. Following this, the homogenate was centrifuged at 2,000 rpm for 10 minutes at 4 °C to recover the protein, which was then quantified using the Lowry method, with BSA as the standard. All protein extracts were stored at −80 °C until further analysis. Proteins were separated using sodium dodecyl sulfate-polyacrylamide gel electrophoresis and transferred onto a polyvinyl difluoride membrane. The membrane was incubated overnight at 4 °C with a primary antibody (phospho-NF-κB p65/NF-κB p65, Santa Cruz) on a rotating table. After the incubation, the membrane was washed 3 times for 10 minutes each with PBS containing 0.5% Tween 20. Next, a horseradish peroxidase-conjugated secondary antibody was applied for 2 hours at 25 °C. After another series of washes (3 times for 10 minutes each with PBS 0.5% Tween 20), the chemiluminescence signal was detected using an EC detection reagent (Thermo Fisher) and captured with the BioSpectrum 415 Image Acquisition System (UVP). The chemiluminescence data were analyzed using ImageJ software version 1.50a.

### Docking studies and molecular dynamics simulations

To complement and strengthen in vitro experiments, in silico–guided docking and molecular dynamics (MD) simulations were performed for predicting potential interactions of cannabidiol and rosiglitazone (RGZ) with PPAR-γ. First, ethanol solvent sites were identified via the MDMix method^33^ using the crystal structure of human PPAR-γ complexed with RGZ (PDB ID:5ycp), from which RGZ was removed to expose the ligand-binding pocket. Guided docking of cannabidiol was directed toward residues L330, I341, and L255 within the ligand-binding pocket using rDock. Previous preparation of the protein encompassed modeling missing residues with Modeller version 10.4^34^ and protonation at physiological pH 7.4 with the aid of PDBfixer.^35^ MD simulations were conducted using the AMBER 22 package in combination with the FF19SB force field.^36^^,^^37^ Relative binding free energy was calculated by the Molecular Mechanics/Generalized-Born Surface Area (MM/GBSA) method^38^ applied over 1,000 frames from the last 50 ns of the MD simulations. Further details including detailed parameters and used packages are presented in the Supplemental Appendix.

### Statistical analysis

Statistical data are presented as mean ± SEM (SEM) of at least 3 independent experiments. Comparisons between groups were made by unpaired Student's *t*-test, while >2 groups were compared using 1- or 2-way analysis of variance followed by Tukey's post-hoc test for multiple pairwise comparisons. Non-normally distributed data were compared using Kruskal-Wallis test followed by Dunn's post hoc test. A *P* value <0.05 was considered statistically significant. Data processing, graphs, and statistical analysis were performed with GraphPad Prism (version 8).

## Results

### Cannabidiol prevents cardiac pathological structural changes in mice with HF

**Figure 1:**
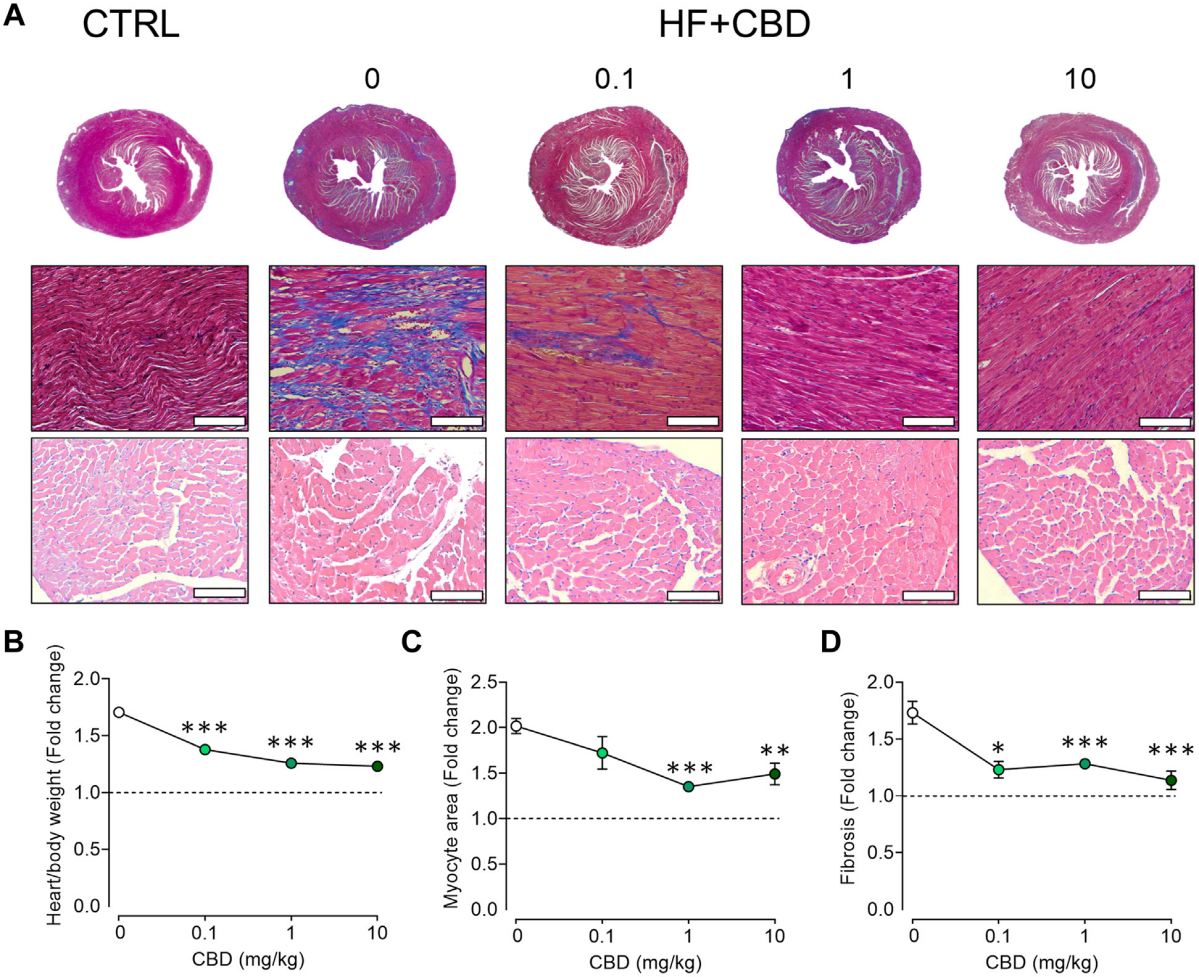
Antifibrotic and Antihypertrophic Effects of CBD in the Heart of Mice With HF (A) Representative micrographs of cardiac tissue as a function of cannadibiol (CBD) dosage (in mg/kg): Masson’s Trichrome stain to visualize fibrotic changes in upper row, 1.25×, and middle row 10×; hematoxylin and eosin stain for cardiac myocyte area assessment is presented in the bottom row, 10×. Quantification of dose-dependent effect of CBD in: (B) heart/body weight, normalized to control group (dotted line); (C) myocyte area; and (D) fibrotic index. The scale bars in representative micrographs correspond to 100 μm. Data are presented as mean ± SEM; n = 4-20; analyzed by 1-way analysis of variance test with post hoc Tukey multiple comparisons test. ∗*P <* 0.05, ∗∗*P <* 0.01, and ∗∗∗*P <* 0.001 compared with 0 mg/kg of CBD. CBD = cannabidiol; CTRL = control; HF = heart failure.

To assess the effects of cannabidiol in vivo, we employed a 2-hit HF mouse model using L-NAME and ANGII administration, and after progression for 28 days, cardiac remodeling was confirmed (Figure 1). All further mice-related experiments were performed at the endpoint of 28 days of the HF mouse model. The structural effects of cannabidiol on the HF heart were evaluated by administering it at a dose rate of 0.1, 1, or 10 mg/kg every third day. Representative slides of Mason’s trichrome and H & E showed an inverse relationship between cardiac fibrosis and the myocyte area with increasing cannabidiol dose (Figure 1A). Indeed, the heart-to-body weight ratio was reduced proportional to the cannabidiol dose (Figure 1B) (*P* [vs 0] = <0.001, <0.001, and <0.001 for 0.1, 1, and 10 μmol/L), with similar results in both myocyte area (Figure 1C) (*P* [vs 0] = <0.001 and 0.002 for 1 and 10 μmol/L) and cardiac fibrosis (Figure 1D) (*P* [vs 0] = <0.014, <0.001, and <0.001 for 0.1, 1, and 10 μmol/L), preventing structural remodeling and hypertrophy. Notably, administration of cannabidiol to healthy mice did not result in structural changes (Supplemental Figures 2A and 2C), in agreement with previous studies.^19^ Cannabidiol exhibited a dose-dependent effect in preventing cardiac remodeling and inflammation. Considering that the most effective cannabidiol dose was 1 mg/kg, this concentration was selected for further animal studies.

### Cannabidiol preserves heart function in HF mice

**Figure 2:**
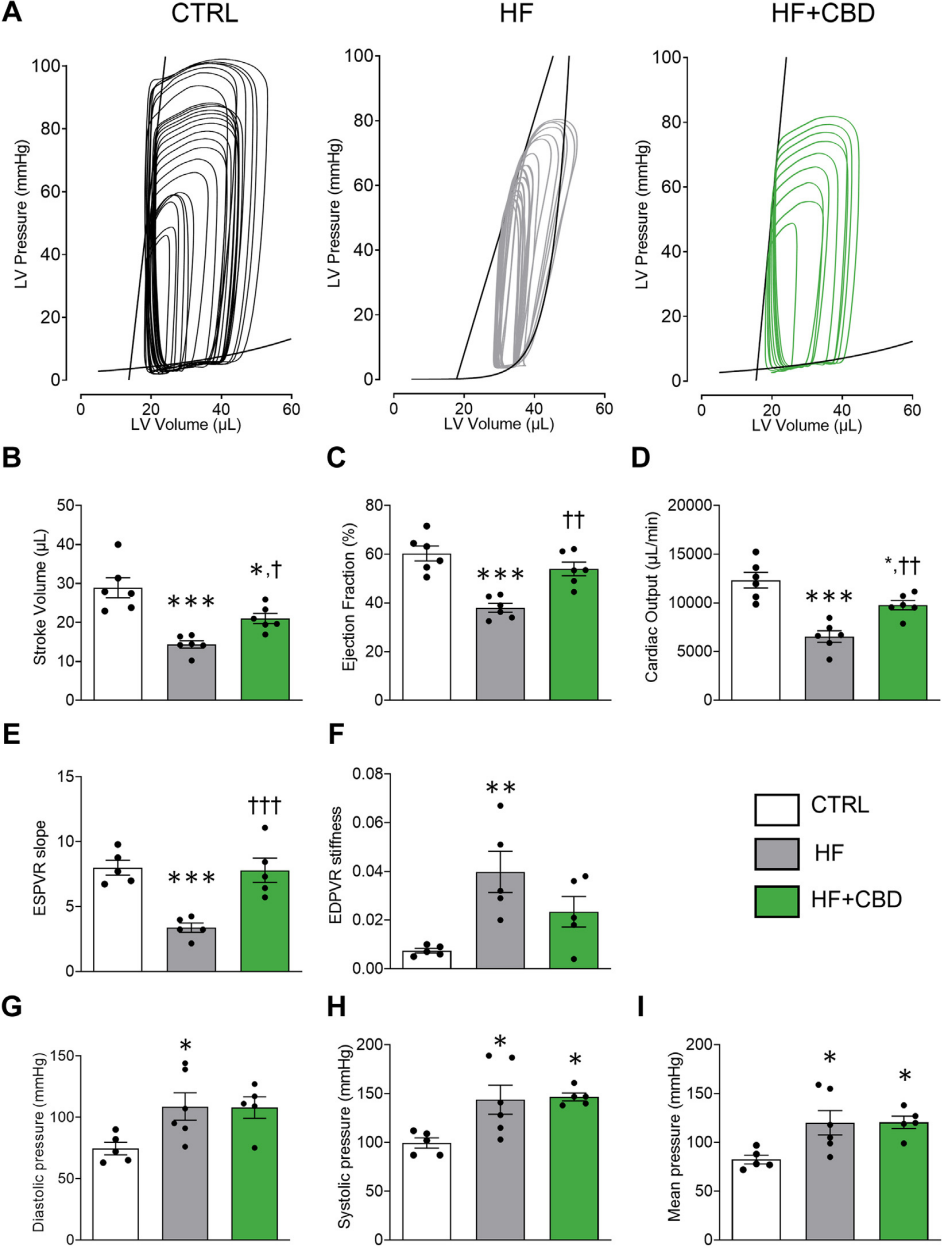
CBD Protects Against Cardiac Dysfunction in Mice With HF (A) Representative images of pressure-volume loop recordings with preload impediment by transiently occluding the inferior vena cava, of vehicle (CTRL), HF, or HF + CBD infusion for 28 days, and the end-diastolic pressure-volume relationship (EDPVR) slope and end-systolic pressure-volume relationship (ESPVR) slope originated (black lines), respectively. First loop represents steady-state loop. Hemodynamic parameters determined from steady-state pressure-volume loop analysis: (B) stroke volume; (C) ejection fraction; (D) cardiac output; (E) ESPVR; and (F) EDPVR slope values for each group. Pooled data of: (G) diastolic, (H) systolic, and (I) mean blood pressure in CTRL, HF, and HF + CBD mice groups. The group of HF + CBD was treated with 1 mg/kg of CBD. Data are presented as mean ± SEM; n = 5-6; dots represent individual values; analyzed by 1-way analysis of variance test with post hoc Tukey multiple comparisons test; ∗*P* < 0.05, ∗∗*P* < 0.01, and ∗∗∗*P* < 0.001 compared with CTRL, †*P* < 0.05, ††*P* < 0.01, †††*P* < 0.001 compared with HF. Abbreviations as in Figure 1.

To investigate the protective effect of cannabidiol in HF, cardiac function was assessed using PV loop measurements. This approach enabled the evaluation of key hemodynamic parameters and revealed how cannabidiol mitigates HF-induced cardiac dysfunction. Representative images of the PV loops during inferior vena cava occlusion are reported in Figure 2A, where the first loop from right to left represents the steady state. We observed a profound impairment in the HF group in heart steady-state hemodynamic parameters, with statistically associated reductions in stroke volume (SV) and ejection fraction (EF), a reduction in cardiac output (CO), induced by the increment of the end-systolic pressure-volume relationship (ESPVR), and an end-diastolic pressure-volume relationship (EDPVR) (Figures 2B to 2F) (SV: *P <* 0.001; EF: *P <* 0.001; CO: *P <* 0.001; ESPVR: *P =* 0.001; EDPVR: *P =* 0.007), as well as diastolic, systolic, and mean blood pressures (Figures 2G to 2I) (diastolic pressure: *P =* 0.047; systolic pressure: *P =* 0.024; mean pressure: *P =* 0.029). Importantly, treatment with cannabidiol was able to offer protection from HF deleterious effect on steady-state parameters (Figures 2A to 2D) (EF: *P =* 0.002; CO: *P =* 0.007; SV: *P =* 0.040), where EF in the HF + CBD group was not statistically different to the control group.

In accordance with these data, cannabidiol was able to counteract systolic dysfunction induced by HF after inferior vena cava occlusion (Figure 2E) (ESPVR: *P <* 0.001). ESPVR slope analysis showed that HF induced systolic dysfunction by acting likely through ANGII, a negative inotropic agent that reduces contractility or systolic performance, as shown by a slope shifted to the right, with a value reduced compared with the control group, which was prevented by cannabidiol (Figure 2E), suggesting that chronic HF-induced systolic dysfunction may implicate a deleterious effect on the molecular mechanisms responsible for cardiomyocyte stimulus-contraction coupling and that cannabidiol could be acting at that level to prevent cellular dysfunction. Moreover, the diastolic function EDPVR slope analysis showed that HF increased ventricular compliance (stiffness), which was partially prevented by cannabidiol (Figure 2F), without statistical significance. This indicates that with HF-induced chronic systolic dysfunction, the EDPVR shifts as the ventricle responds to anatomic tissue remodeling, thus increasing ventricular compliance. Nevertheless, diastolic, systolic, and mean pressure did not vary between the HF and HF+CBD groups (Figures 2G to 2I). In addition, cannabidiol did not elicit any cardiac function changes when administered to healthy mice (Supplemental Figures 2B and 2D). Altogether, these results demonstrate the potential therapeutic effect of cannabidiol in preventing the deleterious effect of HF cardiac performance, with possible implications of a protective effect at the level of the molecular mechanisms of stimulus-contraction coupling and/or tissue remodeling signaling.

### Cannabidiol blocks remodeling, inflammation, and apoptosis in mice with HF

**Figure 3:**
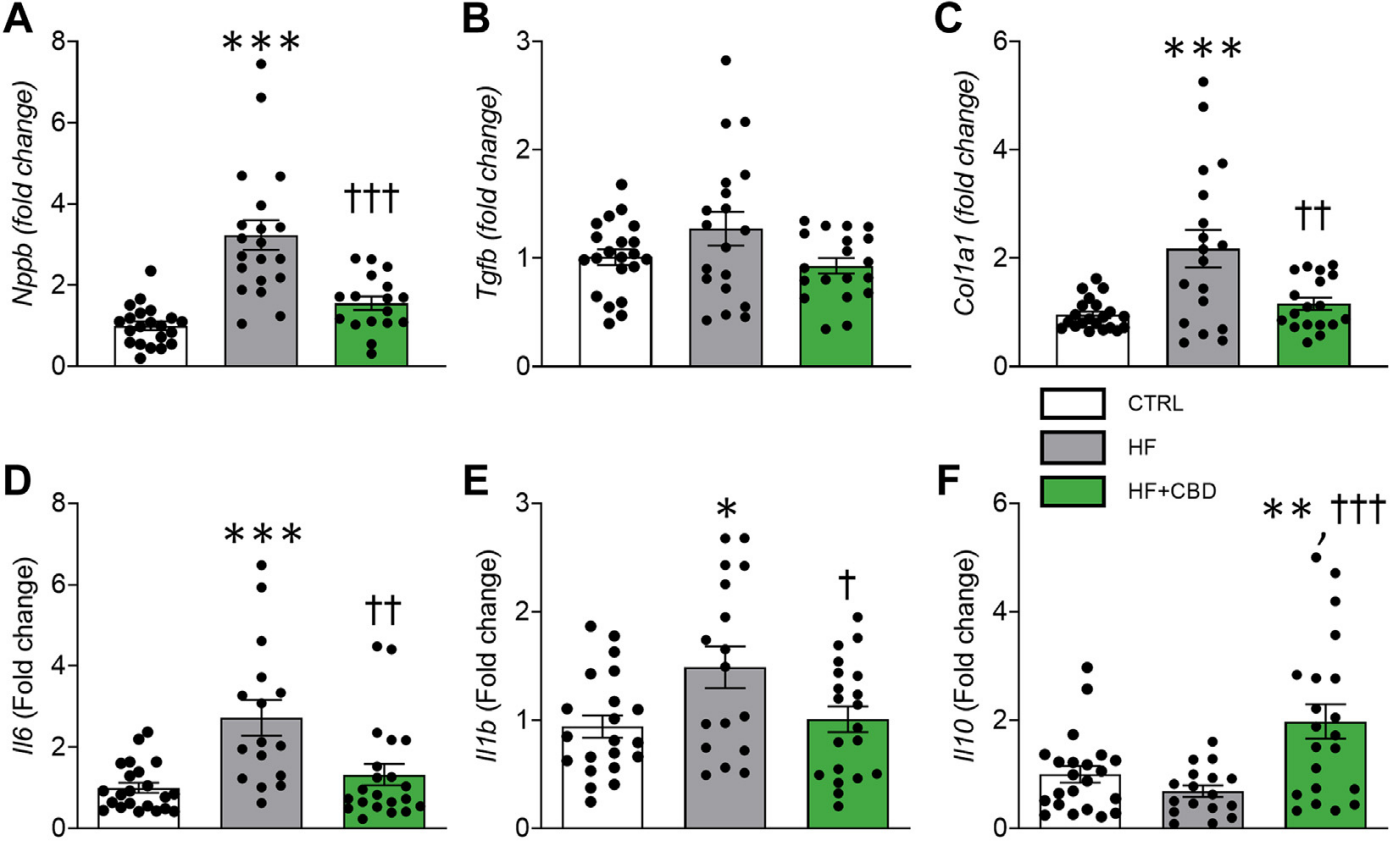
The Remodeling, Immunomodulatory, and Antiapoptotic Effect of Cannabidiol in Heart Tissue of Mice With HF Gene expression of cardiac remodeling markers: (A) BNP (Nppb gene), (B) TGF-β (Tgfb1 gene), and (C) Col1a (Col1a1 gene), normalized to control group (dotted line). GAPDH was used as housekeeper gene. Gene expression of cytokines in cardiac tissue: (D) IL-6 (*Il6* gene), (E) IL-1β (*Il1b* gene), (F) IL-10 (*IL10* gene). The group of HF + CBD was treated with 1 mg/kg of CBD. Data are presented as mean ± SEM; n = 3-20; dots represent individual values; analyzed by 1-way analysis of variance test with post hoc Tukey multiple comparisons test. ∗*P <* 0.05, ∗∗*P <* 0.01, and ∗∗∗*P <* 0.001 compared with CTRL. †*P* < 0.05, ††*P* < 0.01, †††*P* < 0.001 compared with HF. Abbreviations as in Figure 1.

To examine the impact of cannabidiol on cardiac remodeling, inflammation, and apoptosis in HF, molecular markers were assessed. This analysis aimed to determine how it may block pathological changes in tissue structure, inflammatory cytokine release, and cell death, thus potentially offering protection against HF-induced cardiac damage. Cardiac structural changes were consistent with B-type natriuretic peptide (BNP) reduction, a marker determining the severity of hemodynamic dysfunction/cardiac failing, presenting a statistically associated increase in HF (Figure 3A) (*P <* 0.001), which was halted with cannabidiol administration. In addition, tissue growth factor beta (TGF-β) showed a tendency to increase in HF and diminish with cannabidiol administration (Figure 3B), albeit not statistically associated. Furthermore, extracellular matrix deposition showed a similar outcome, as cannabidiol treatment prevented collagen type 1a (Col1a) overexpression in HF (Figure 3C) *(P =* 0.007). Healthy mice administered with cannabidiol did not result in statistically associated molecular marker changes (Supplemental Figure 2E).

Besides cardiac remodeling, HF is also characterized by an inflammatory process that includes the increased release of proinflammatory cytokines.^39^ The effects of cannabidiol on the inflammatory response and apoptosis were assessed in the animal model caused by the observed antifibrotic and antihypertrophic effects. Mice with HF showed inflammation in the heart statistically associated with increases of proinflammatory cytokines including IL-6, and -1β levels, with a statistically not associated decrease in the anti-inflammatory cytokine IL-10 (Figures 3D to 3F), in agreement with previous reports.^23^ Cannabidiol treatment managed to prevent this phenomenon by preventing increase of cytokines IL-6 and -1β, while importantly increasing IL-10 expression (Figures 3D to 3F) (*Il6: P =* 0.003; *Il1b*: *P =* 0.048; *Il10*: *P <* 0.001). No changes were observed with TNF-α expression (data not shown). No statistically associated cytokines were observed in healthy mice administered cannabidiol (Supplemental Figure 2F).

### Cannabidiol averts cellular energetics failure

**Figure 4:**
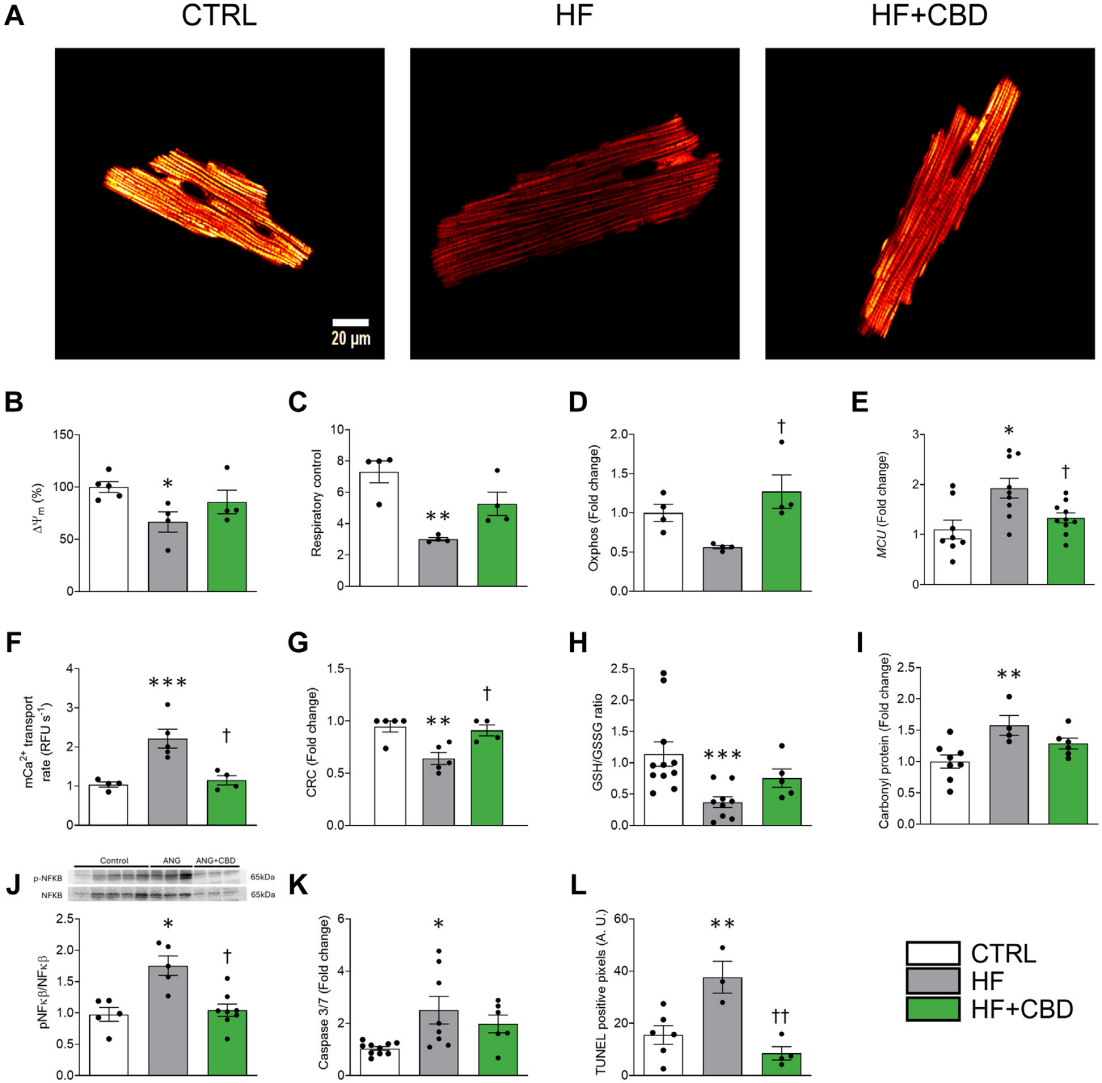
CBD Prevents Mitochondrial Dysfunction and Oxidative Stress Increase in Cardiomyocytes and Cardiac Tissue (A) Representative images of ΔΨm, and (B) its quantification from isolated LV myocytes. From cardiomyocyte-isolated mitochondria: (C) respiratory control, (D) state 3 (phosphorylating respiration). From cardiomyocytes: (E) mitochondrial calcium uniporter (MCU) expression, (F) Mitochondrial Ca^2+^ transport rate, (G) Ca^2+^ retention capacity (CRC). From heart homogenates: (H) total protein carbonylation, and (I) reduced glutathione (GSH) and oxidized glutathione (GSSG) ratio (GSH/GSSG ratio), (J) ratio of phosphorylated NFκβ to NFκβ protein expression, (K) caspase 3/7 activity assay, (L) terminal deoxynucleotidyltransferase-mediated dUTP-biotin nick end labeling (TUNEL) assay. The group of HF+CBD was treated with 1 mg/kg of CBD. Data are presented as mean ± SEM; n = 4-11; dots represent individual animals. Data of (B, D-L) were normalized to their control group; analyzed by 1-way analysis of variance test with post hoc Tukey multiple comparisons test. ∗*P <* 0.05, ∗∗*P <* 0.01, and ∗∗∗*P <* 0.001 compared with CTRL. †*P* < 0.05, ††*P* < 0.01 compared with HF. Abbreviations as in Figure 1.

**Figure 5:**
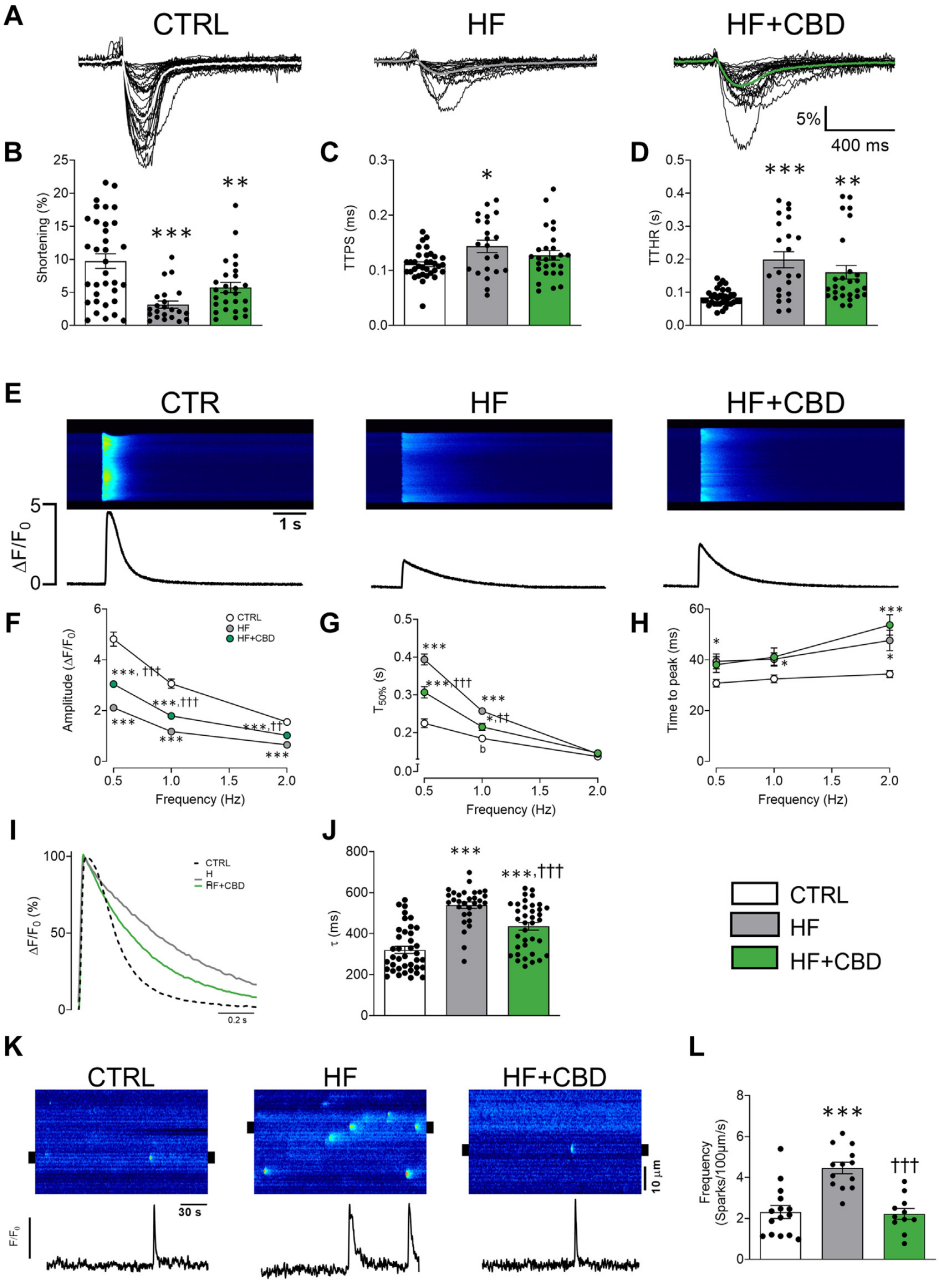
CBD Improves Cardiomyocyte Cellular Contractility and Ca^2+^ Handling in Mice With HF Representative profile of (A) cellular shortening, (B) maximal shortening, (C) time to peak shortening (TTPS), and (D) time to half relaxation (TTHR) at 0.5 Hz. (E) Representative images and fluorescence profile of Ca^2+^ transient at 0.5 Hz, (F) pooled data from Ca^2+^ transient amplitude, (G) T_50%_, and (H) time to peak at 0.5, 1, and 2 Hz. (I) Representative fluorescence profile and (J) pooled data of decay tau (τ) at 0.5 Hz. (K) Representative line scan from treated groups, below are shown line profiles from 2-μm regions of the selected spark (black marks in line scan images), and (L) pooled data of Ca^2+^ spark frequency. Control (CTRL) (dotted line), heart failure (HF) (gray line), and HF treated with CBD (HF + CBD) (green line). The group of HF + CBD was treated with 1 mg/kg of CBD. Data are presented as mean ± SEM; n = 22-39; dots represent individual cells; Kruskal-Wallis test with post hoc Dunn multiple comparisons test (B-D, J, L) or 2-way analysis of variance test with post hoc Tukey multiple comparisons test (F-H). ∗*P <* 0.05, ∗∗*P <* 0.01, and ∗∗∗*P <* 0.001 compared with CTRL. †*P <* 0.05, ††*P <* 0.01, and †††*P <* 0.001 compared with HF. Abbreviations as in Figure 1.

To examine the effects of cannabidiol on cellular energetics in HF, the mitochondrial function and oxidative stress markers in cardiac myocytes were assessed. By evaluating parameters such as mitochondrial membrane potential (ΔΨm) and respiratory control, the aim was to determine how cannabidiol mitigates HF-induced energy production failure and preserves antioxidant capacity, preventing cellular bioenergetic dysfunction. The cardiac function, represented by ECC coupling, involves high energy-dependent mechanisms, such as cellular relaxation and intracellular Ca^2+^ uptake, in which proper mitochondrial function is paramount to maintain ATP production and sustain this process. The HF model exhibited compromised mitochondrial function, as the ΔΨm, the electrochemical force to synthesize ATP, was decreased in myocytes isolated from the HF group compared with those isolated from the control group (Figures 4A and 4B) *(P* = 0.046), possibly compromising ATP production. However, in the HF + CBD group, the decrease in ΔΨm was not statistically different from the control group (Figures 4A and 4B). To better explain the loss of ΔΨm in HF cardiac mitochondria, the respiratory function of mitochondria in isolated organelles was analyzed. A marked reduction of the respiratory control (RC) was observed (Figure 5C) *(P* = 0.002), but there was a statistically not associated decrease in oxidative phosphorylation (Figure 5D). These alterations were concomitant to a statistically associated increase in the MCU gene expression (Figure 5E) *(P* = 0.005), along with an increase in the intramitochondrial Ca^2+^ transport and CRC (Figures 5F and 5G) *(P =* 0.002 and 0.005, respectively). None of these parameters showed a statistically associated change between the HF + CBD group and the control group (Figures 5C to 5G). Cardiac myocyte cellular stress increased in the HF group, as shown by a decreased GSH/GSSG ratio (Figure 5H) *(P =* 0.005) and augmented protein carbonylation (Figure 5I) *(P =* 0.010). Cannabidiol administration to the HF group resulted in statistically not associated reduction of oxidative stress, partially mitigating the changes of the GSH/GSSG ratio (Figure 5H) and protein carbonylation (Figure 5I). In addition, phosphorylated NF-κβ had a statistically associated increase (Figure 5J) *(P =* 0.019).

HF caused an increase in cellular death, as shown by statistically associated higher levels of executioner caspases 3/7 as well as higher apoptosis (Figures 3K and 3L). These results suggest that HF cardiac myocytes are affected by mitochondrial dysfunction and dysregulation of the antioxidant capacity of the cell; however, the cannabidiol administration (HF + CBD group) can prevent these changes. Healthy mice treated with cannabidiol did not show statistically associated changes to cardiomyocytes’ bioenergetic and oxidative status (Supplemental Figures 3C and 3F to 3H).

### Cannabidiol maintains excitation-contraction-energetic coupling in mice with HF

To investigate how cannabidiol preserves excitation-contraction-energetic coupling in HF, cardiac myocytes were isolated to evaluate intracellular Ca^2+^ dynamics and cell contraction (Figure 5A). This approach allowed the assessment of the ability of cannabidiol to prevent HF-induced impairments in cellular contractility and Ca^2+^ handling, offering insights into its protective effects on heart function at the cellular level. The HF group showed compromised cellular contractility with a decrease in maximal shortening (Figure 5B) (*P <* 0.001), an increase in both phases of the contraction-relaxation cycle, TTPS (Figure 5C) *(P =* 0.012), and more importantly, TTHR (Figure 5D) (*P <* 0.001). The cannabidiol-treated HF group, compared with the HF group, did not show statistically associated changes in the maximum contractility and did not statistically decrease TTPS or TTHR (Figures 5B to 5D). Correspondingly, the HF group showed altered intracellular Ca^2+^ dynamics, decreasing transient amplitude and increasing T_50%_ compared with the control group, whereas cannabidiol treatment partially prevented both changes (Figures 5F and 5G) (amplitude: *P =* <0.001, <0.001, and 0.002 at 0.5, 1, and 2 Hz; T_50%_: *P =* <0.001 and 0.002 at 0.5 and 1 Hz). The time to peak was statistically associated with an increase in the HF group compared with the control group, and treatment with cannabidiol did not result in statistically associated differences to the HF group (Figure 5H). Because cytosolic Ca^2+^ removal determines cellular relaxation, further examination of transient decay was performed. The HF group showed a statistically associated increase of Ca^2+^ recapture rate (τ), which was partially reversed by cannabidiol (Figure 5J) (*P <* 0.001). Thus, the results indicate improved cell contraction and intracellular Ca^2+^ handling with cannabidiol administration in the HF model. To further explore the effects of cannabidiol on HF and cardiac myocyte Ca^2+^ handling, basal Ca^2+^ dynamics were evaluated through Ca^2+^ sparks production (Figure 5K). The HF group showed statistically associated hyperactivity of the ryanodine receptors (RyR), because spark frequency was increased in comparison to the control group,^40^ while the HF + CBD group prevented such increase (*P <* 0.001), keeping it without statistical difference to the control group (Figure 5L). Spark amplitude did not change between groups, which might indicate a lack of change in reticulum sarcoplasmic Ca^2+^ content between groups.^41^ The cardiomyocyte function from healthy mice administered with cannabidiol was not statistically different (Supplemental Figures 3A, 3B, 3D, and 3E). These results suggest that in addition to cardiac contractility, HF modulates Ca^2+^ handling; however, cannabidiol partially prevents such effects.

### Cannabidiol-induced antihypertrophic effects are partially mediated by the PPAR-γ pathway and preservation of mitochondrial function

To investigate the potential pathways involved in the cardioprotective effects of cannabidiol, ventricular cardiomyoblasts (H9c2 cell line) were treated with ANGII to induce hypertrophy (Supplemental Figures 4A to 4N). This study aimed to determine how cannabidiol prevents hypertrophy and mitochondrial dysfunction, focusing on the role of the PPAR-γ pathway and its impact on mitochondrial oxidative stress and Ca^2+^ overload. As observed in the animal model, ANGII increased mitochondrial reactive oxygen species (mROS), consequently reducing ΔΨm and oxidative phosphorylation capacity. Mitochondrial Ca^2+^ content ([Ca^2+^]_m_) was increased, as well as [Ca^2+^]_m_ uptake rate and MCU expression, with a concomitant increase in mitochondrial fragility. By contrast, cannabidiol administration prevented hypertrophy and pathological remodeling in a dose-dependent manner, with doses ranging from 0.001 up to 1 μmol/L, (Supplemental Figures 4A and 4B) (*P* [vs 0] = 0.79, 0.007, 0.005, and 0.012 for 0.001, 0.01, 0.1, and 1 μmol/L, respectively), resulting in surface area on par with control cells at the highest doses. Gene expression of remodeling markers BNP, TGFβ, and Col1a were exacerbated in hypertrophied cells, and cannabidiol administration reduced these remodeling markers in a dose-dependent manner (Supplemental Figures 4C to 4E) (Npbp: *P* [vs 0] = 0.87, 0.006, 0.004, and <0.001 for 0.001, 0.01, 0.1, and 1 μmol/L, respectively; Tgfb1: *P* [vs 0] = 0.11, 0.003, 0.004, and 0.003 for 0.001, 0.01, 0.1, and 1 μmol/L, respectively; Col1a: *P* [vs 0] = 0.93, 0.019, 0.006, and 0.004 for 0.001, 0.01, 0.1, and 1 μmol/L, respectively), reaching levels similar to those of the control cells at the highest doses, in agreement with the data observed in cardiomyocytes. Cannabidiol also reduced dose-dependent mROS overproduction in hypertrophied cells (Supplemental Figures 4F and 4G) (P [vs 0] = 0.083, 0.052, 0.039, and 0.020 for 0.001, 0.01, 0.1, and 1, respectively, for Supplemental Figure 4G). In addition, ANGII was statistically associated with reductions to ΔΨm, RC, and oxidative phosphorylation (Supplemental Figures 4H to 4J). These bioenergetic changes were statistically associated with an increase in MCU gene expression, mitochondrial Ca^2+^ (mCa^2+^) transport rate, reduced CRC, and an overall increase in [Ca^2+^]_m_ (Supplemental Figures 4K to 4N). Administration of cannabidiol (ANGII + CBD group) resulted in statistically not different outcomes in these bioenergetic and Ca^2+^-related mitochondrial gene and dynamics, except for a statistically associated partial prevention in loss of Oxphos (Supplemental Figure 4J). The results suggest that in this cellular model, the antihypertrophic effect of cannabidiol is linked to the reduction of mitochondrial oxidative stress, which is modulated by the mitochondrial Ca^2+^ overload.

**Figure 6:**
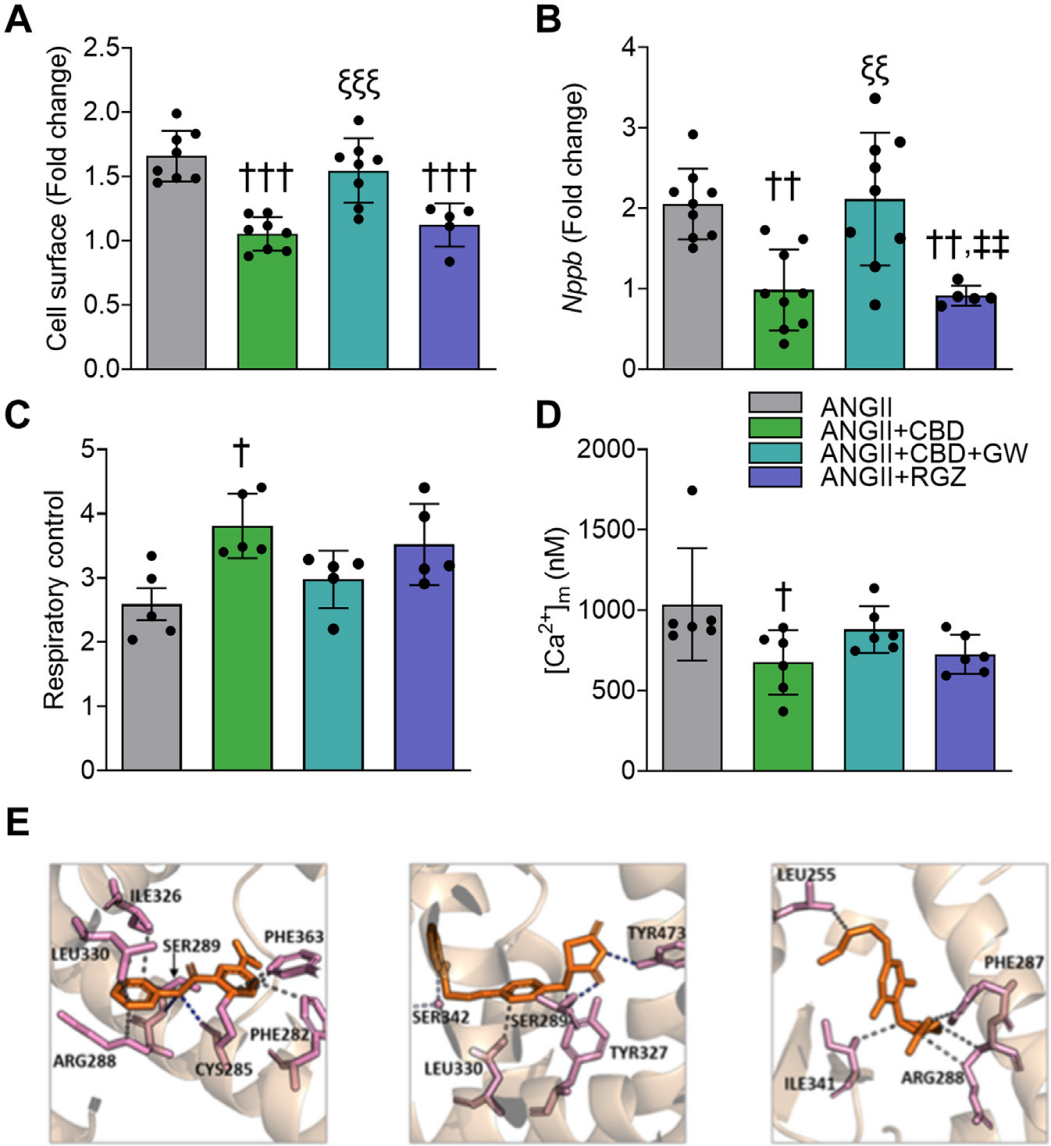
Activation of PPAR-γ Mimics the Anti-Inflammatory and Antiremodeling Effects of CBD in Hypertrophic H9c2 Cells, and CBD Interacts Similarly to Other Agonists With PPAR-γ (A) Cell surface area. (B) BNP (Nppb gene). (C) Respiratory control. (D) Mitochondrial Ca^2+^ content [Ca^2+^]_m_. The groups of angiotensin II (ANGII) + CBD and ANGII + CBD + GW9662 (GW) in A-D were treated with 0.01 μmol/L of CBD. Data are presented as mean ± SEM; n = 5-6; dots represent individual values; analyzed by 1-way analysis of variance test with post hoc Tukey multiple comparisons test. †*P <* 0.05, ††*P <* 0.01, and †††*P <* 0.001 compared with ANGII. *P <* 0.001 compared with ANG+CBD, *P <* 0.01 compared with ANGII + CBD + GW. (E) Crystallographic structure of peroxisome proliferator-activated receptor γ (PPAR-γ)-GW (PDB ID: 6md1) (left), PPAR-γ-rosiglitazone (RGZ) (PDB ID: 5ycp) (middle), and the predicted docking pose of CBD bound to PPAR-γ (right). Abbreviations as in Figure 1.

Even if cannabidiol has shown low affinity for the cannabinoid CB1 and CB2 receptors,^42^ evidence suggests that cannabidiol interacts at low concentrations with CB1 either as an antagonist^43^ or a negative allosteric modulator of CB1,^44^ and with CB2 as a partial agonist.^45^^,^^46^ In this regard, under hypertrophy conditions, Rimonabant (blocker of CB1 receptor) and SR141 (blocker of CB2 receptor) did not statistically reduce the antihypertrophic effect of cannabidiol (Supplemental Figure 5). We also assessed the contribution of PPAR-γ pathways, which have been proposed molecular targets of cannabidiol.^47^ GW9662 (GW) was used as an antagonist of PPAR-γ, and RGZ was used as a PPAR-γ agonist. Administration of GW along with cannabidiol under ANGII stimulation resulted in abolition of the antihypertrophic effect of cannabidiol (Figure 6A) (*P <* 0.001), increasing BNP expression (Figure 6B) *(P =* 0.001) and Col1A expression (Supplemental Figure 6A) *(P =* 0.02) and an increase on protein content (Supplemental Figure 6B) *(P =* 0.043). RGZ administration mimicked the cannabidiol effects of preventing increased cell surface area (Figure 6A) (*P <* 0.001) and BNP expression (Figure 6B) (*P* [vs ANGII] = 0.007). Such changes elicited the question of whether mitochondrial dysfunction could be prevented through the activation of PPAR-γ. To further elucidate the mitochondrial status within these outcomes, the RC and [Ca^2+^]_m_ were measured, revealing a statistically not associated tendency for mitochondrial uncoupling and increased [Ca^2+^]_m_ in the ANGII + CBD + GW group (Figures 6C and 6D), but a statistically associated increase in MCU expression (Supplemental Figure 6C) *(P =* 0.023), with RZG showing no statistical difference to the effect of CBD in either RC or [Ca^2+^]_m_ (Figures 6C and 6D). Healthy cells treated with CBD, GW, or RZG did not exhibit statistically associated alterations in either morphology, remodeling, mitochondrial Ca^2+^ transport, and bioenergetics (Supplemental Figures 6D to 6H). These changes are suggestive of the suppression of mCa^2+^ overload in the ANGII+CBD and ANGII+RGZ groups and its abrogation by the addition of GW.

### Cannabidiol might activate PPAR-γ by direct interaction on the canonical ligand-binding pocket

To elucidate the interaction of cannabidiol with PPAR-γ docking and MD studies were conducted. The interactions between PPAR-γ (Supplemental Figure 7A) and the molecules were performed (Supplemental Figure 7B, Supplemental Tables 2 and 3). Cannabidiol was docked into the canonical ligand-binding pocket (residues L330, I341, and L255) using guided docking. The obtained docked pose with PPAR-γ revealed that cannabidiol stabilized in the same orthosteric site as RGZ through nonbonding interactions of hydrophobic character (Figure 6E, Supplemental Figure 7C, Supplemental Table 4). Furthermore, MD simulations at 150 ns reached equilibrium (root mean square deviation <0.2 nm) (Supplemental Figure 7D) revealed RGZ and cannabidiol ligands ideally buried in the hydrophobic pocket with PPAR-γ and exhibiting similar binding energies (Supplemental Figure 7E, Supplemental Table 4). In particular, there were no significant changes in the RGZ conformation inside the pocket before or after MD simulations. By contrast, the cannabidiol conformation was allocated near the activation function-2 helix in PPAR-γ. These data suggest a potential interaction between cannabidiol and PPAR-γ.

## Discussion

HF physiopathology, marked by contractile dysfunction, cardiac remodeling, inflammation, and characterized by Ca^2+^ mishandling, is anchored by ROS-driven mitochondrial dysfunction through alterations of Ca^2+^ handling, which impairs mitochondrial excitation-contraction-energetic coupling.^5^, ^6^, ^7^, ^8^^,^^48^ This knowledge offers an opportunity for therapeutic development to improve HF management by addressing such an altered cardiac energetic state. Here, cannabidiol was demonstrated to protect mitochondrial energetic status and ROS production, controlling pathological remodeling and inflammation in HF. Additionally, in a surrogate model of ventricular cardiomyoblasts, we determined that PPAR-γ signaling could be involved in cannabidiol cardioprotective activity.

### Therapeutic use of cannabidiol in HF

Recently, a growing number of preclinical studies have stressed the potential of cannabidiol to attenuate cardiac dysfunction in the setting of cardiovascular disease. Even acute interventions with low doses of cannabidiol (50-100 μg/kg) can effectively limit the size of an ischemic lesion and reduce arrhythmia associated with myocardial infarction in rats and rabbits.^14^^,^^15^^,^^49^ Furthermore, in a chronic disease scenario, daily applications of cannabidiol (10-20 mg/kg) prevented and even reverted LV dysfunction in murine models of autoimmune and diabetic cardiomyopathy. In these cases, cannabidiol substantially improved systolic and diastolic performance and ameliorated myocardial stiffness (EDPVR).^19^^,^^20^ In the present study, 1 mg/kg of cannabidiol delivered every third day for 4 weeks was determined to be sufficient for counteracting, to a large extent, the functional decline of the LV in HF driven by pressure overload. Notably, the degree of improvement in contractile performance was comparable to that reported elsewhere.^19^^,^^20^ Although we are aware that higher doses of cannabidiol have been employed before (10-20 mg/kg), we did not observe any further benefit when a higher dose of 10 mg/kg was used in terms of myocyte hypertrophy (Figure 1), BNP expression, or Col1a deposition (data not shown). Thus, in this particular context, the therapeutic effect exerted by cannabidiol appeared to plateau near the selected dose of 1 mg/kg. In this regard, it is worth adding that we employed a synthetic formulation of high-purity cannabidiol in the present study, whereas in previous reports, cannabidiol was extracted from hashish.^19^^,^^20^ Apart from containing other bioactive cannabinoids that may confound the interpretation of the results,^50^ the cannabidiol concentration could be lower and closer to what we report here. It might also be possible that the effective dose of cannabidiol is disorder-specific.

### Cardioprotective effect of cannabidiol in cardiac remodeling and inflammation

To a great extent, functional improvement can be attributed to preserving myocardium structural integrity. In particular, cannabidiol mediates a significant antifibrotic action, switching off the TGF-β signaling pathway and thereby limiting collagen and fibronectin deposition within the myocardium.^19^^,^^51^ In agreement, we found a dose-dependent decrease of TGF-β and Col1A gene expression, which paralleled a reduction in the fibrotic index of Masson’s trichrome-stained ventricular sections and may underlie the partial recovery of LV compliance. Moreover, because profibrotic signaling is often secondary to an inflammatory response, it is noteworthy that cannabidiol not only diminished the expression of classically proinflammatory cytokines but also increased the anti-inflammatory cytokine IL-10 at the transcriptional level, even above control baseline values.

Regardless of the precise mechanism, a decrease of proinflammatory cytokines (namely IL-1β, IL-6, TNF-α) concomitant with an increase of IL-10, typically an anti-inflammatory cytokine, after CBD administration is a well-documented effect that has been reported in systemic as well as in local inflammation in animal models.^52^^,^^53^ In particular, a broad anti-inflammatory effect in the heart has been observed before in preclinical models of autoimmune and methamphetamine-induced myocarditis, although the molecular pathways implicated remain elusive.^20^^,^^54^ Such an anti-inflammatory effect of cannabidiol in the heart has been formerly ascribed to a reduction in mononuclear infiltration^13^^,^^20^ and the local inhibition of NF-κβ signaling.^17^^,^^19^ In turn, reduced inflammation may result from cannabidiol’s potent redox balance modulation, which encompasses a direct interaction with reactive species and metal ions and the activation of the endogenous antioxidant systems in the cell.^55^

### Cannabidiol, mitochondrial bioenergetics, and the redox system

Indeed, a recurrent finding in a wide range of cardiovascular disease models is that cannabidiol reduces oxidative and nitrosative stress within the myocardium.^17^, ^18^, ^19^, ^20^^,^^56^ In accordance, we observed that in HF, cannabidiol mitigated oxidative stress, measured as protein carbonyl content, and improved the GSH/GSSG ratio in cardiac tissue. Although we cannot rule out the contribution of cannabidiol over iNOS or NADPH oxidase, our data suggest that cannabidiol mainly acts by curbing ROS of mitochondrial origin (Supplemental Figure 4). We want to emphasize that, in line with a previous report indicating that the administration of cannabidiol did not exert anti-hypertensive activity,^56^ we found that cannabidiol did not influence the blood pressure of HF animals in the current study (Figures 2G to GI). Therefore, our findings cannot be explained in terms of relieved pressure overload but perhaps by direct interaction of cannabidiol with a molecular target in the cardiomyocyte, namely, the PPAR-γ nuclear receptor.

Alternatively, it has been claimed that cannabidiol directly interacts with proteins in the mitochondrial membranes.^10^^,^^11^ Indeed, the extensively documented neuroprotective effect of cannabidiol has been partially attributed to the improvement of mitochondrial bioenergetics, which favors cell survival.^57^, ^58^, ^59^ Similarly, cannabidiol boosted mitochondrial biogenesis in the myocardium and enhanced the activity of respiratory complexes, limiting the cell death associated with doxorubicin chemotherapy.^18^ Intriguingly, in hippocampal neurons, cannabidiol worked as a protector against several insults, presumably by lowering mCa^2+^ and thereby preventing the opening of the mPTP.^11^^,^^12^

### Effects of cannabidiol on oxidative stress and mCa^2+^

Cardiac relaxation is a cellular process highly dependent on an adequate mitochondrial ATP supply.^3^ In this sense, mitochondrial dysfunction appears when mCa^2+^ transport is stimulated (Figure 4F, Supplemental Figure 4L), which can lead to mCa^2+^ overload and mPTP opening,^60^ as seen in the HF group in this study (Figure 4G, Supplemental Figure 4M and 4N). Such increased access of Ca^2+^ into the mitochondria is consistent with an increase of the MCU expression in both HF cardiomyocytes (Figure 4E) and hypertrophied H9c2 cells (Supplemental Figure 4K), and has been previously reported in heart failure models and patient samples.^8^ Consequently, mROS leaking out of the mitochondria and into the cytosol has numerous effects. It can initiate a signaling cascade by oxidizing the inhibiting proteins of NF-κβ, as previously described,^61^ promoting inflammation (Figure 3). It can also reach nearby structures and alter protein function, such as RyR.^62^ The oxidant environment prevailing in the failing cardiomyocyte seems to promote both phosphorylation and oxidation of RyR2^63^ and calmodulin,^64^ which translates into RyR2 leakiness and consequent contractile impairment and increased susceptibility to arrhythmia. We could attribute the effects of cannabidiol over SR Ca^2+^ release, at least in part, to the strong antioxidant properties of cannabidiol, which could have ameliorated the redox state of the RyR2 making it less prone to spontaneous diastolic openings, which we did observe as a reduction in spark frequency (Figure 5K and 5L). Moreover, cannabidiol appeared to have improved Ca^2+^ reuptake during the decaying phase of the Ca^2+^ transient (Figure 5G). Again, we could conjecture that this is a consequence of the powerful nonspecific antioxidant effects of cannabidiol over SERCA2a, which activity is known to be deeply affected in oxidant conditions.^65^ Another possible target of mROS-induced oxidation is sarcoendoplasmic reticulum calcium ATPase, which reduces its activity and increases the time needed to recapture the Ca^2+^ every beat,^66^ as evidenced by a greater T50 and τ (Figure 5). These targets not only cause more inefficient Ca^2+^ handling, but may also contribute to worsening the mCa^2+^ overload and initiating a vicious cycle.^60^ Notably, mCa^2+^ overload has been linked to the development of hypertrophy^67^ and remodeling.^49^ Preventing mitochondrial Ca^2+^ overload or mROS production has proven effective in reducing cardiac hypertrophy and pathological remodeling.^49^^,^^68^^,^^69^

Here, we demonstrated that cannabidiol administration has a similar effect as an inhibitor of mitochondrial Ca^2+^ transport, as it reduces mitochondrial Ca^2+^ overload and mROS production and prevents NF-κβ activation and inflammation. The exact mechanism of action of cannabidiol has yet to be determined. Initially, this was proposed to occur through a CB1 antagonist effect, although the idea was later dismissed.^14^ In this regard, eg, in a lymphoblastic leukemia cell line that is highly sensitive to cannabidiol treatment, its effect does not depend on CB1/2 receptors or plasma membrane Ca^2+^-permeable channels. Instead, cannabidiol directly targets mitochondria and alters their capacity to handle Ca^2+^, modifying mitochondrial Ca^2+^ overload and reducing mPTP formation.^10^ Another possibility is the inhibition of the cannabinoid receptor GPR55,^70^ which has been demonstrated to be relevant in cardiac tissue, as it may contribute to myocardial ischemia/reperfusion injury^71^ and may regulate Ca^2+^ release in neonatal cardiomyocytes.^72^ However, conflicting evidence exists regarding this; eg, knockout mice lacking GPR55 develop ventricular remodeling, systolic dysfunction, and a reduced contractile reserve.^73^ In the context of myocardial infarction, GPR55 knockout mice developed worse LV dilation and a more extensive infarct area.^74^ Nonetheless, this pathway cannot be completely defined, and more studies are needed to determine the role of GPR55 in cardiovascular diseases and its therapeutic potential with cannabidiol inhibition.

### Activation of PPAR-γ by cannabidiol

Another signaling pathway proposed for cannabidiol is the activation of PPAR-γ.^75^ Following previously reported data, inhibiting PPAR-γ with GW prevents the protection conferred by cannabidiol administration (Figures 6A to 6D), including increases in cellular remodeling and MCU expression (Supplemental Figures 6A and 6C). These findings are further supported by docking and molecular dynamics studies in which cannabidiol is demonstrated to have a potential interaction with PPAR-γ (Figure 6E).

**Figure 7:**
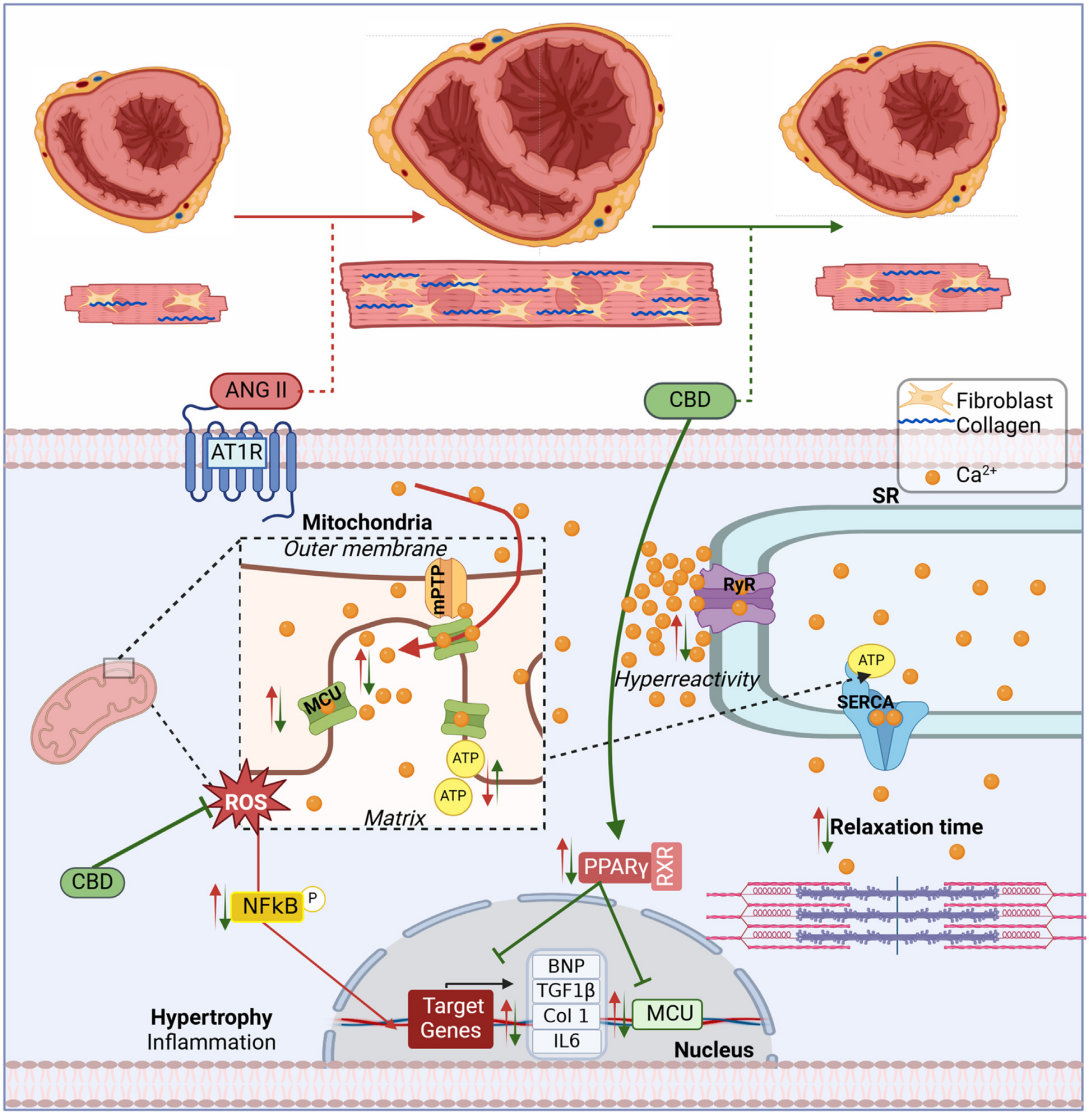
Proposed Mechanism of CBD Antihypertrophic Effect ANGII induces pathological remodeling and decreases cardiac performance, leading to hypertrophy, cardiac fibrosis, increased reactive oxygen species (ROS) production, mitochondrial dysfunction, and elevated proinflammatory and prohypertrophic factors and biomarkers in an HF with reduced ejection fraction model (effects represented by red arrows). In contrast, cannabidiol (CBD), depicted by green arrows, demonstrates cardioprotective effects by attenuating cardiac fibrosis, enhancing sarcoplasmic reticulum Ca^2+^ uptake, maintaining mitochondrial function, and preserving redox balance. Additionally, in vitro studies showed that CBD activates the PPARγ pathway, reducing the expression of remodeling biomarkers and MCU. mPTP = mitochondrial permeability transition pore; other abbreviations as in Figures 1, 4, and 6.

Finally, to integrate a more detailed explanation of the possible mechanisms through which CBD preserves bioenergetics and relieve oxidative stress, we looked into the results of MCU expression and PPAR-γ modulation on hypertrophied H9c2 cells. That is because an increased MCU expression can easily explain the increased Ca^2+^ content and thus the observed increased oxidative stress.^49^ First of all, and to our surprise, CBD preserved MCU expression similar to control levels (Figure 4E). Next, because PPAR-γ is thought to drive the cellular pathway of CBD, we sought to elucidate whether the effect of CBD on MCU expression was caused by this receptor. A known PPAR-γ agonist, RGZ, was used as an activator of PPAR-γ. Interestingly, RGZ had the same effect in ANGII-treated cells, preventing cellular hypertrophy, while using GW9662, a PPAR-γ antagonist, abolished the protective effect of CBD (Figures 6A and 6B, Supplemental Figure 6C). Because augmented MCU activity, with a consequential mitochondrial Ca^2+^ overload, is reported to drive an increase in mitochondrial ROS production with subsequent mitochondrial dysfunction, we infer that mitochondrial preservation and the reduced oxidative stress seen with CBD treatment is at least partially caused by a modulated MCU expression by CBD through PPAR-γ activation, which protects the mitochondria against Ca^2+^ overload. These experiments suggest that the effects of cannabidiol observed in the studied HF model are at least partially caused by the activation of the PPAR-γ signaling pathway. In all, these results have inspired a translational effort, as reflected by the development of clinical trials aiming to assess the potential of cannabidiol in HF and cardiac dysfunction (NCT04615949, NCT05494788, NCT05180240, NCT06708299). Finally, Figure 7 presents the summary of the findings in this study.

### Study limitations

Our data support that cannabidiol exerts cardioprotection in the HF mouse model by limiting fibrosis and hypertrophy while sustaining ejection fraction and cardiac output. This was possible because of preservation of cardiomyocyte cell shortening, sarcoplasmic reticulum Ca^2+^ uptake, mitochondrial function, and redox balance. In addition, analysis of cannabidiol effects on an ANGII hypertrophy model in ventricular cardiac myoblast suggests PPAR-γ activation as a pathway of cardioprotection. Nevertheless, limitations to this study are as follows: 1) the findings have not been corroborated in human cardiac tissue samples, which limits the direct translation of the presented results; 2) the PPAR-γ pathway was found in a cell culture of ventricular cardiac myoblast, a surrogate model of a cardiomyocyte, limiting at this point the strength and relevance of the finding in the scope of adult cardiomyocytes; 3) in the current study, cannabidiol was administered at the onset of the ANGII supply for the development of the HF mouse model, and its effects remain to be studied once the model is established; and 4) this study was only performed in male mice, mainly because studying both sexes simultaneously can complicate understanding of CBD pathways caused by sex hormone and hepatic metabolism interactions. Thus, this initial focus on male mice allows a foundational understanding before exploring sex differences, given reports of female mice exhibiting resistance to develop heart failure compared with male mice.^76^

## Conclusions

This study demonstrated that cannabidiol offers cardioprotection in a HF mouse model induced by L-NAME and ANGII administration. The results showed improved cardiac function and reduced cardiac hypertrophy, remodeling, inflammation, and cell death. In cardiomyocytes from the HF model, cannabidiol restored cell shortening, which was linked to improved calcium Ca^2+^ handling.

Additionally, it helped preserve cellular oxidative status, mitochondrial bioenergetics, and notably, modulated mCa^2+^ overload by affecting MCU expression. This suggests that the cardioprotective effects of cannabidiol are caused by the preservation of excitation-contraction-energetic coupling. The identified cellular mechanisms through which cannabidiol exerts its cardioprotective effects include reducing oxidative stress and the activation of PPAR-γ, which helps prevent mitochondrial dysfunction by decreasing MCU hyperactivity.

Perspectives **COMPETENCY IN MEDICAL KNOWLEDGE:** The outcomes of this study add toward a deeper understanding of the cellular and molecular basis of HF, particularly in regard to cardiac hypertrophy, Ca^2+^ dynamics, and mitochondrial function of cardiomyocytes.

**TRANSLATIONAL OUTLOOK:** This study contributes to the knowledge of a novel therapy based on cannabidiol on the pathophysiology of HF, which is supported by preclinical data. Here, we described that cardioprotection exerted by cannabidiol on a HF mouse model was caused by the attenuation of cardiac fibrosis and hypertrophy along with improved ejection fraction and cardiac output. This was achieved, in the cardiomyocyte, by preservation of cell shortening, sarcoplasmic reticulum Ca^2+^ uptake, mitochondrial function, and redox balance, with data supporting the role of a PPAR-γ–dependent mechanism. This study suggests promising therapeutic results of cannabidiol used in the clinical field of HF treatment. In this regard, these results have inspired a translational effort to assess its effects in HF and cardiac dysfunction.

## Funding Support and Author Disclosures

This work was supported in part by Cardiol Therapeutics, and CONACYT Grants 256577, 258197, Fronteras de la Ciencia grant 0682, and Ciencia Básica grants A1-S-23901 and A1-S-43883. Drs Garcia-Rivas, Lozano, and Torre-Amione are partners of Nano 4 Heart, SC. Mr Bolton works for Cardiol Therapeutics Inc. Dr Torre-Amione is a board member of Cardiol Therapeutics Inc. All other authors have reported that they have no relationships relevant to the contents of this paper to disclose.
