# Supplementary material for: Cannabidiol Prevents Heart Failure Dysfunction and Remodeling Through Preservation of Mitochondrial Function and Calcium Handling
Source: JACC Basic Transl Sci. 2025 Feb 19;10(6):800–21. doi: 10.1016/j.jacbts.2024.12.009 (PMC12230462; doi:10.1016/j.jacbts.2024.12.009)
Supplement: Supplemental Material [file mmc1.docx]

**Supplemental Appendix**

**Page**

**Supplemental Methods …………………………………………………………………………. 2**

**Supplemental Tables .…………………………………………………………………............... 6**

**Supplemental Table 1 …………………………………………………………………............... 6**

**Supplemental Table 2 …………………………………………………………………............... 6**

**Supplemental Table 3 …………………………………………………………………............... 8**

**Supplemental Table 4 …………………………………………………………………............... 9**

**Supplemental Figures .……………………………………………………………………........ 10**

**Supplemental Figure 1 ……………………………………………………………………........ 10**

**Supplemental Figure 2 ……………………………………………………………………........ 12**

**Supplemental Figure 3 ……………………………………………………………………........ 14**

**Supplemental Figure 4 …………………………………………………………………............ 16**

**Supplemental Figure 5 …………………………………………………………………............ 18**

**Supplemental Figure 6 …………………………………………………………………............ 19**

**Supplemental Figure 7 …………………………………………………………………............ 20**

**References …………………………………………………………………………………............ 22**

**Supplemental methods**

**Docking studies and molecular dynamics simulations.**

**Identification of solvent sites**

The MDMix method was used to determine ethanol solvent sites, as described elsewhere (1). The PPARγ-RGZ crystal structure, from which the ligand molecule was removed, was used to run five MD replicas of 20 ns each. The protein was embedded in a box of water/ethanol 80/20% v/v. Cartesian restrictions of 0.1 kcal/mol Å_2_ were applied on the protein-heavy atoms, following the running conditions in the MD simulation section. Density maps for probe atoms were obtained by constructing a static mesh of grids over the entire simulation box and counting the occurrence of probe atoms in each grid throughout trajectories. The observed appearance was converted into binding free energy using the Boltzmann relationship, considering the observed probe atom distribution and the expected distribution in a bulk solvent at 1.0 M. Solvent sites were filtered by imposing an energy threshold of 1 kcal/mol. Moreover, solvent sites were used as pharmacophoric elements using rDock to dock cannabidiol into the canonical ligand binding pocket (residues L330, I341, and L255).

**Protein and ligand preparation**

The crystal structure of a human PPARγ ligand binding domain solved with rosiglitazone (PPAR-γ-RGZ) was obtained from the Protein Data Bank (PDB ID: 5ycp) (2). Meanwhile, a docking model was used for the complex with cannabidiol (PPARγ-CBD). The first 9 residues from the crystal structure in PPARγ are missing. The first residue in the protein was capped with ACE groups to avoid artificial terminal charges. Moreover, segments 262-272 (corresponding to the W loop) were not solved. The peptide derived from the human steroid receptor coactivator-1 was removed. The residues were modeled using Modeller v10.4 (3). The proteins were protonated at pH 7.4 using PDBfixer (4). Using AMBER's tLeap, each protein was placed in a truncated octahedral box, initially spanning 10 Å further from the solute in each direction, and then solvated with an OPC water model. The hydrogen mass repartitioning scheme was implemented using the ParmEd (https://github.com/ParmEd/ParmEd), which allows a 2-fs time step integration. The systems were geometrically optimized using the steepest descent algorithm for 5000 cycles to adjust the solvent orientation and eliminate local clashes. Systems were simulated using periodic boundary conditions. Ewald sums (grid spacing of 1 Å), as implemented in the PMEMD module, were used for treating long-range electrostatic interactions with a 9 Å cutoff for direct interactions (5). A similar cutoff was also applied to Lennard-Jones interactions. The SHAKE algorithm was applied to fix any bond involving hydrogen atoms (6). Unless otherwise stated, no other constraints were used. Coordinates were saved every 10 ps. RGZ and cannabidiol topologies and parameter files were generated with the antechamber suite and the general Amber force field (GAFF2) using AM1-BCC atomic charges (7). Cannabidiol and RGZ parameters are listed in Supplemental tables 2 and 3, respectively.

**MD simulations and analysis**

MD simulations were performed using the AMBER 22 suite in conjunction with the FF19SB force field (8,9). Before MD production, each molecular construct was subjected to the steepest descent energy minimization. Afterward, the same was subjected to two equilibration steps in canonical (NVT) and isobaric-isothermal (NPT) assemblies. Initial velocities were assigned to get a 150 K distribution, gradually increasing to 310 K in 0.8 ns in the NVT ensemble. All systems were further equilibrated for 1 ns at 310 K in the NPT ensemble. After equilibration, the MD production was performed in an NPT ensemble under the Langevin thermostat (collision frequency of about 4 ps-1) and the Berendsen barostat (pressure relaxation time of 2 ps) at 310 K for 150 ns with five replicas for each system. MD trajectories and interatomic distances were analyzed with CPPTRAJ; root-mean-square deviation (RMSD) graphics were generated with matplotlib.

**Relative binding free energy calculations**

One thousand frames from the last 50 ns of MD simulations were used to perform the relative binding free energy calculations. The energetic calculations were performed under the MM/GBSA (Molecular Mechanics/Generalized-Born Surface Area) method implemented in the Amber package and calculated with MMPBSA.py script (10) following the standard equations:


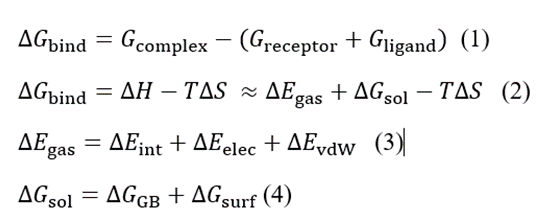


The relative binding free energies (ΔGbind) can be estimated as the arithmetic sum of the free energies for the receptor and ligand relative to the total free energy of the complex (eq. 1). However, the calculated binding free energies were approached as the sum of the gas-phase interaction energy and solvation free energy without considering entropy effects (eq. 2). First, the changes for the internal energy change (ΔEint) were canceled because the structures of complex, receptor, and ligand were extracted from the same trajectory. Thus, the gas-phase interaction energy (ΔEgas) between the receptor and the ligand was approached as the sum of electrostatic (ΔEelec) and van der Waals (ΔEvdW) interaction energies. Meanwhile, the solvation-free energy (ΔGsol) was approached as the contribution of the polar and non-polar energy terms. The polar solvation energy (ΔGGB) was calculated by using the GB (Generalized Born) model, and the non-polar contribution was calculated based on the solvent-accessible surface area (ΔGsurf). A value of 80 was used for the solvent dielectric constant. The Amber input parameter igb was set to 2 for an ionic strength of 150 mM.

**Supplemental tables**

Supplemental Table 1. List of primers used for gene assessment by qPCR.

| **Gene** **name** | **Encoded protein** | **Forward primer 5’-3'** | **Reverse primer 5’-3'** |
| --- | --- | --- | --- |
| *Nppb* | Brain natriuretic peptide B | CTCCAGAACAATCCACGAT | CTTGAACTATGTGCCATCTTG |
| *Tgfb1* | Transforming growth factor beta 1 | AGGGCTACCATGCCAACTTC | CCACGTAGTAGACGATGGGC |
| *Col1a1* | Collagen I A1 | GACTGTCCCAACCCCCAAAA | CTTGGGTCCCTCGACTCCTA |
| *Il6* | Interleukin 6 | CTTGCTGGTGGATGTTC | GGAGTGCTGCTTGGATG |
| *Il1b* | Interleukin 1 beta | CAACCAACAAGTGATATTCTCCATG | GATCCACACTCTCCAGCTGCA |
| *Il10* | Interleukin 10 | CCAAGGTGTCTACAAGGCCA | GTCCTGTCTAGGTCCTGGAGT |
| *Tnfa* | Tumor necrosis factor alpha | CCAGTGTGGGAAGCTGTCTT | AAGCAAAAGAGGAGGCAACA |

Supplemental Table 2. Atom types and partial charges used for cannabidiol and its final structure. The van der Waals parameters and bonded terms are those associated with these particular atom types in the Amber forcefield.

| **Atom name** | **Atom**  **type** | **Partial charge** | **Atom name** | **Atom**  **type** | **Partial**  **charge** | **Atom name** | **Atom type** | **Partial charge** | **Atom name** | **Atom type** | **Partial charge** |
| --- | --- | --- | --- | --- | --- | --- | --- | --- | --- | --- | --- |
| C11 | c3 | -0.0921 | C14 | c2 | -0.1154 | HC4 | hc | 0.0377 | HC15 | hc | 0.0512 |
| C10 | c3 | -0.0854 | C15 | c3 | -0.0372 | HC5 | hc | 0.0377 | HC16 | hc | 0.0477 |
| C9 | c3 | -0.0764 | C16 | c3 | -0.0754 | HC6 | hc | 0.0427 | HC17 | ha | 0.1100 |
| C8 | c3 | -0.0734 | C17 | c3 | -0.0145 | HC7 | hc | 0.0427 | HC18 | ha | 0.1100 |
| C7 | c3 | -0.0401 | C18 | c2 | -0.0994 | HC8 | hc | 0.0507 | HC19 | hc | 0.0430 |
| C5 | ca | 0.0027 | C19 | c2 | -0.2280 | HC9 | hc | 0.0507 | HC20 | hc | 0.0430 |
| C4 | ca | -0.2520 | C20 | c3 | -0.0689 | HC | ha | 0.1345 | HC21 | hc | 0.0430 |
| C3 | ca | 0.1641 | C21 | c3 | -0.0549 | HC1 | ha | 0.1345 | HC22 | hc | 0.0380 |
| C2 | ca | -0.1133 | O2 | oh | -0.4936 | HC10 | hc | 0.0727 | HC23 | hc | 0.0380 |
| C1 | ca | 0.1641 | H1 | ho | 0.4175 | HC11 | ha | 0.1210 | HC24 | hc | 0.0380 |
| C6 | ca | -0.2520 | H2 | ho | 0.4175 | HC12 | hc | 0.0432 | HC25 | hc | 0.0317 |
| O1 | oh | -0.4936 | HC2 | hc | 0.0447 | HC13 | hc | 0.0432 | HC26 | hc | 0.0317 |
| C12 | c3 | 0.0458 | HC3 | hc | 0.0447 | HC14 | hc | 0.0512 | HC27 | hc | 0.0317 |
| C13 | c2 | -0.1562 |  |  |  |  |  |  |  |  |  |


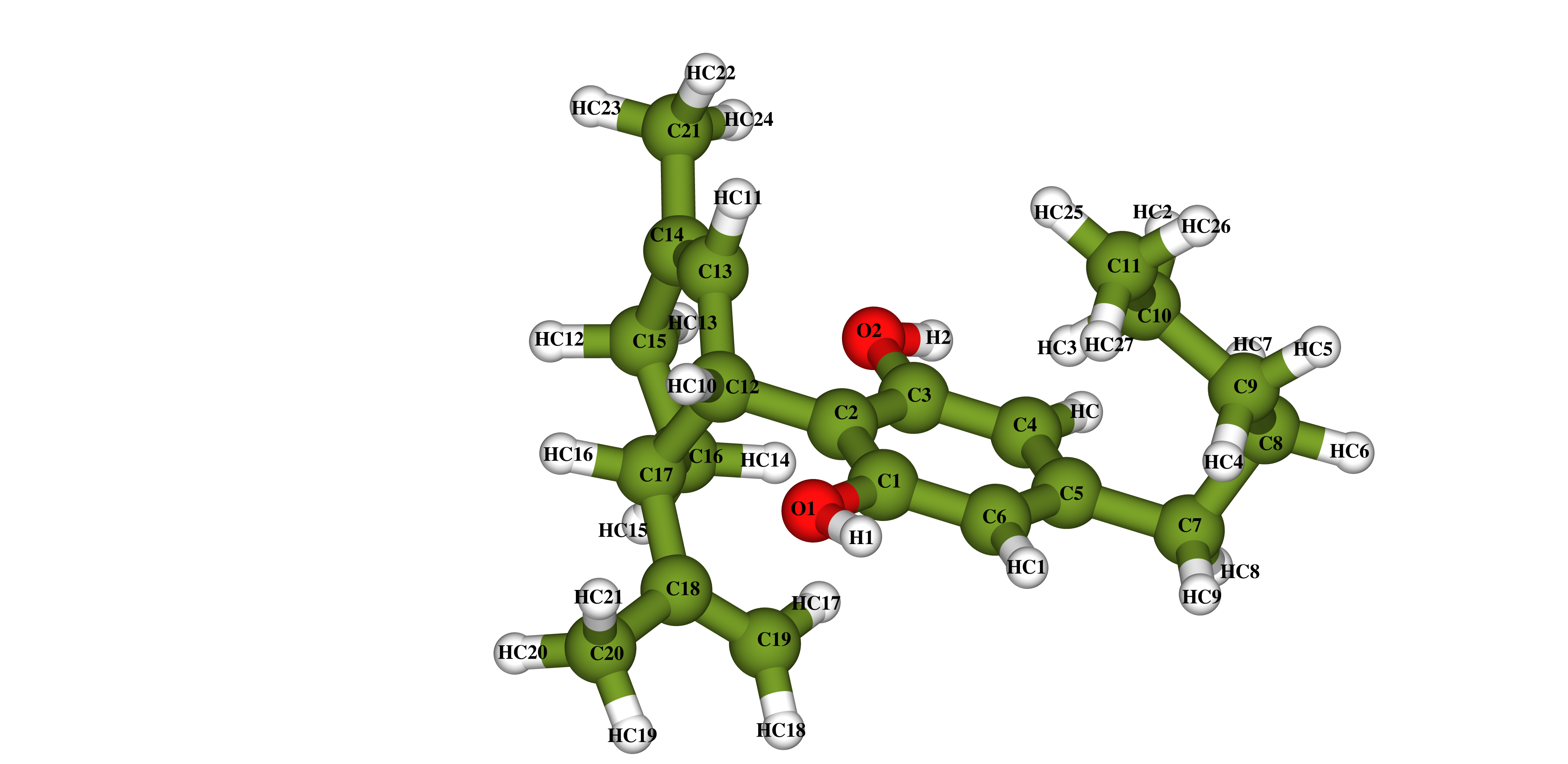


Supplemental Table 3. Atom types and partial charges used for rosiglitazone (RGZ) and its final structure. The van der Waals parameters and bonded terms are those associated with these particular atom types in the Amber forcefield.

| **Atom name** | **Atom**  **type** | **Partial charge** | **Atom name** | **Atom**  **type** | **Partial**  **charge** | **Atom name** | **Atom type** | **Partial charge** | **Atom name** | **Atom type** | **Partial charge** |
| --- | --- | --- | --- | --- | --- | --- | --- | --- | --- | --- | --- |
| S1 | ss | -0.1717 | C7 | ca | -0.1400 | C20 | ca | -0.2900 | H142 | h1 | 0.0600 |
| O2 | o | -0.5515 | C8 | ca | -0.0845 | C21 | ca | -0.0640 | H143 | h1 | 0.0552 |
| O4 | o | -0.5725 | C9 | ca | -0.1810 | C22 | ca | -0.2873 | H152 | h1 | 0.0742 |
| O13 | os | -0.3399 | C10 | ca | 0.1381 | H3 | hn | 0.3545 | H153 | h1 | 0.0742 |
| N3 | ns | -0.6155 | C11 | ca | -0.1810 | H5 | h1 | 0.1257 | H19 | h4 | 0.0181 |
| N16 | nh | -0.7543 | C12 | ca | -0.0845 | H62 | hc | 0.0747 | H20 | ha | 0.1430 |
| N18 | nb | -0.7320 | C14 | c3 | 0.0944 | H63 | hc | 0.0747 | H21 | ha | 0.1360 |
| C2 | c | 0.7141 | C15 | c3 | 0.2078 | H8 | ha | 0.1370 | H22 | ha | 0.1450 |
| C4 | c | 0.6995 | C16 | c3 | 0.1821 | H9 | ha | 0.1470 | H161 | h1 | 0.0454 |
| C5 | c3 | -0.0796 | C17 | ca | 0.6392 | H11 | ha | 0.1470 | H162 | h1 | 0.0454 |
| C6 | c3 | -0.0151 | C19 | ca | 0.4312 | H12 | ha | 0.1370 | H163 | h1 | 0.0454 |


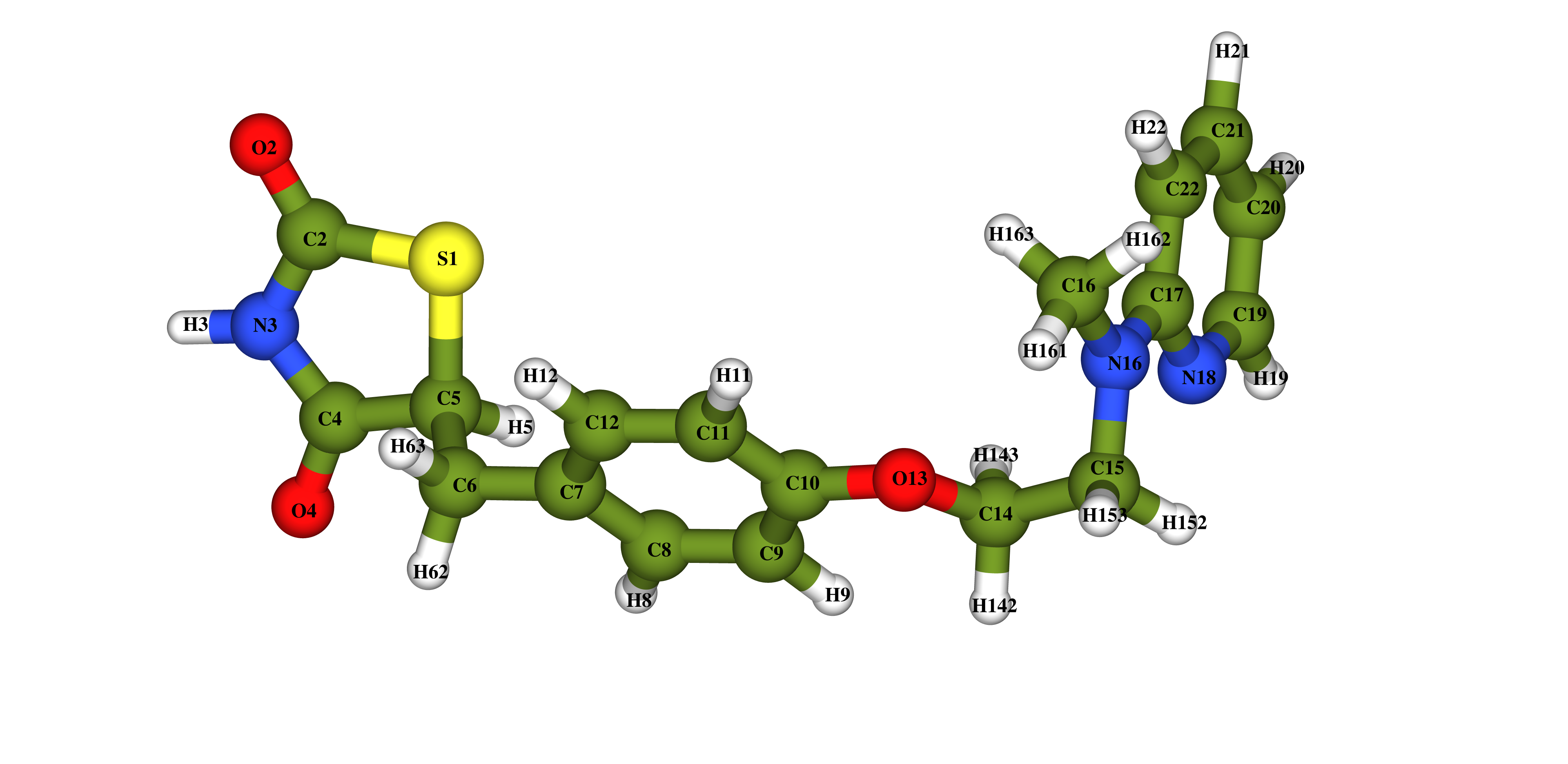


Supplemental Table 4**.** Binding free energy (in kcal/mol) of the residues contributions from the predominant nonbonding interactions of rosiglitazone (RGZ) and cannabidiol (CBD) ligands residing in the orthosteric site for PPARγ.

| **Ligand** | **Residue** | **Interaction type** | **ΔE** |
| --- | --- | --- | --- |
| RGZ | SER289 | H-bond | -1.00 ± 0.4 |
|  | TYR473 | H-bond | -1.34 ± 0.5 |
|  | TYR327 | Hydrophobic | -1.06 ± 0.3 |
|  | ILE326 | Hydrophobic | -1.24 ± 0.3 |
|  | LEU330 | Hydrophobic | -1.64 ± 0.2 |
| CBD | LEU255 | Hydrophobic | -0.67 ± 0.2 |
|  | LEU330 | Hydrophobic | -1.89 ± 0.3 |
|  | ILE341 | Hydrophobic | -1.90 ± 0.3 |

**Supplemental figures**


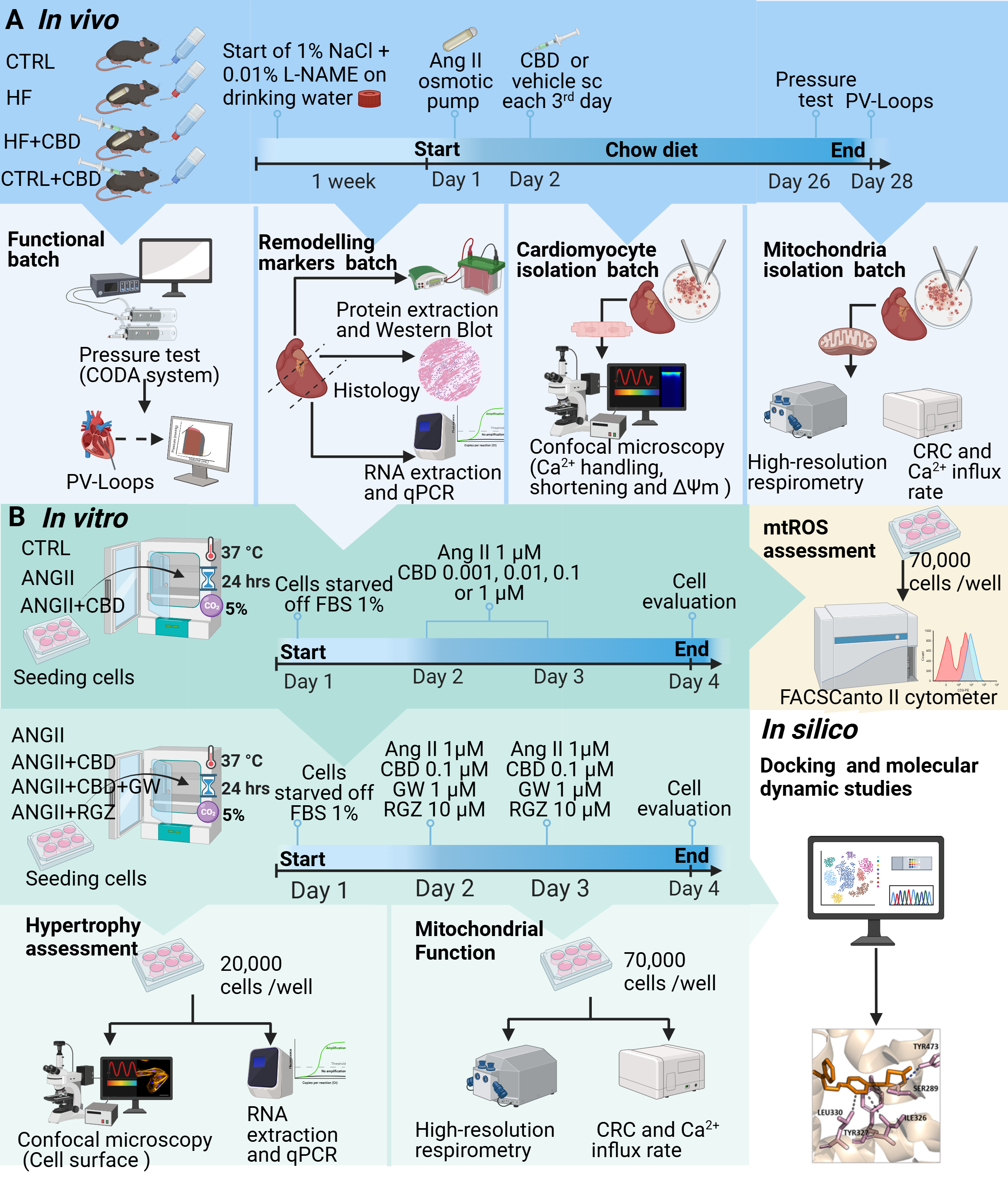


Supplemental Figure 1. Experimental design. (A) Animal Model. Male mice aged 11 weeks were administered water supplemented with 1% NaCl and 0.01% L-NAME for one week. Subsequently, a micro-osmotic pump was surgically implanted subcutaneously in the dorsal area to continuously diffuse ANGII (0.7 mg/kg/day). The control group underwent the same surgical procedure without pump implantation. Following pump implantation, a group of ANGII treated mice received subcutaneous injections of CBD every three days for 28 days, starting on the second day post-implantation. Other groups were injected with an equivalent volume of vehicle. Blood pressure was taken on day 26 after implantation of the pump. On day 28 post implantation, to evaluate hypertrophy and mitochondrial function, one group of animals underwent pressure-volume loop analysis, and their hearts were preserved for protein extraction, RNA analysis, histological sections, and mitochondrial isolation. Another group was used for cardiomyocyte isolation for excitation-contraction-energetic coupling. (B) In vitro model. H9c2 cells were seeded in 6-well plates in DMEM and incubated at 37°C with 5% CO2. After 24 hours, the cells were starved of FBS to 1% and subsequently treated with 1 µM ANGII every 24 hours for two days. The respective treatment groups received 0.001, 0.01, 0.1, and 1 µM cannabidiol, at the end of the treatment period, ROS levels and hypertrophy biomarkers were measured. Following the determination of the optimal CBD concentration, a hypertrophy model was established using a dose of 0.1 µM CBD to assess antihypertrophic effects. Twenty-four hours after the final stimulation, samples were collected for analysis, which included evaluation of mitochondrial function presence of ROS, determination of cell area, extraction of protein and RNA to evaluate hypertrophy, as well as the expression of pro-hypertrophic and pro-inflammatory proteins. Additionally, a molecular docking study was also conducted to predict potential interactions between cannabidiol and rosiglitazone with PPAR-γ.

Supplemental Figure 2. Cannabidiol does not exert hypertrophy, changes in cardiac function, remodeling or inflammation in cardiac tissue of healthy mice. (A) Representative micrographs of cardiac tissue with Masson’s trichrome stain to visualize fibrotic changes (left - 1.25X, middle - 10X) and H&E stain for cardiac myocyte area assessment (right – 10X). (B) Representative images of pressure-volume loop recordings with preload impediment by transiently occluding the inferior vena cava. (C) Heart weight versus body weight, pathomorphological changes of cardiac fibrosis and myocyte area. (D) Stroke volume, ejection fraction, cardiac output, EDP and ESP. (E) Gene expression of remodeling markers BNP, TGF-β, and Col1a. (F) Gene expression pro-inflammatory cytokines TNF-α, IL-1ꞵ, IL-6, and anti-inflammatory cytokine IL-10. The CBD dose was 1 mg/kg. Data are presented as mean ± SEM; n=4-7; dots represent individual values; analyzed by Student's t-test. *p<0.05 compared to the CTRL group.

Supplemental Figure 3. Cannabidiol does not alter cellular contractility, Ca^2+^ handling or mitochondrial function in healthy cardiomyocytes. (A) Representative traces of shortening. (B) Representative traces of Ca^2+^ transients. (C) Representative images of mitochondrial membrane potential (ΔΨ_m_). (D) Shortening, TTPS and TTHR. (E) Amplitude, T_50%_ and TTP. (F) DYm. (G) RC. (H) Oxphos. Data are presented as mean ± SEM; n=4-6; dots represent individual values; analyzed by Student's t-test. *p<0.05 compared to the CTRL group.

Supplemental Figure 4. Cannabidiol prevents pathological remodeling and mitochondrial dysfunction production in hypertrophic H9c2 cells. (A) Representative confocal microscopy images of untreated cells (CTRL), hypertrophic cells (ANGII), and hypertrophic cells treated with 0.1 µM of CBD (ANGII+CBD). (B) Cell surface area of hypertrophic cells as a function of administered CBD. Gene expression of remodeling and inflammatory markers as a function of CBD administration: (C) BNP, (D) TGF-β, and (E) Col1a. (F) Representative histogram of flow cytometry fluorescence of mitochondrial ROS for untreated cells (black dashed line), hypertrophic cells (red color), and hypertrophic cells treated with CBD (green color). (G) Mitochondrial ROS production as a function of CBD administration. (H) ΔΨ_m_. (I) Respiratory control. (J) Oxidative phosphorylation. (K) MCU expression. (L) mCa^2+^ transport velocity in permeabilized cells. (M) CRC. (N) Mitochondrial calcium content [Ca^2+^]_m_, normalized to control group. The group of ANGII+CBD in H-N was treated with 0.01 µM of CBD. Data are presented as mean ± SEM; n=3-5; dots represent individual values; ANOVA test with *post hoc* Tukey multiple comparison test (B-E, G) and Kruskal-Wallis test with *post hoc* Dunn multiple comparisons test. * p<0.05, ** p<0.01, *** p<0.01 compared to either 0 µM CBD or CTRL group; ^†^ p<0.05 compared to ANGII.

Supplemental Figure 5. Antihypertrophic effect of cannabidiol is not modulated by CB1/CB2 receptors in H9c2 cells. Cell surface area of hypertrophic cells, where inhibitors of CB1 (Rimonabant) and CB2 (SR14) did not alter the antihypertrophic effect observed with CBD administration. The groups of ANGII+CBD, ANGII+Rimo+CBD, and ANGII+SR14+CBD in B were treated with 0.01 µM of CBD and 1 µM of either Rimo or SR14. Data are presented as mean ± SEM; n=3; dots represent individual values; One way ANOVA test with *post hoc* Tukey multiple comparison test. ^***^ p<0.001 vs CTRL, ^†^ p<0.05 vs ANGII.

Supplemental Figure 6. Effects of CBD and PPAR-γ antagonist (GW) and agonist (RGZ) on H9c2 cells. On hypertrophic H9c2 cells GW prevents action of CBD: (A) Col 1a, (B) Protein, (C) MCU. On healthy H9c2 cells neither CBD nor PPAR-γ antagonist/agonist exert any effect: (D) Cell surface, (E) BNP, (F) Respiratory control, (G) Oxphos, (H) [Ca2+]m. Data are presented as mean ± SEM; n=3-5; dots represent individual values; analyzed by one way ANOVA test with *post hoc* Tukey multiple comparisons test. ^a^ p<0.05, compared to ANGII; ^ξ^ p<0.05 compared to ANGII+CBD.

**
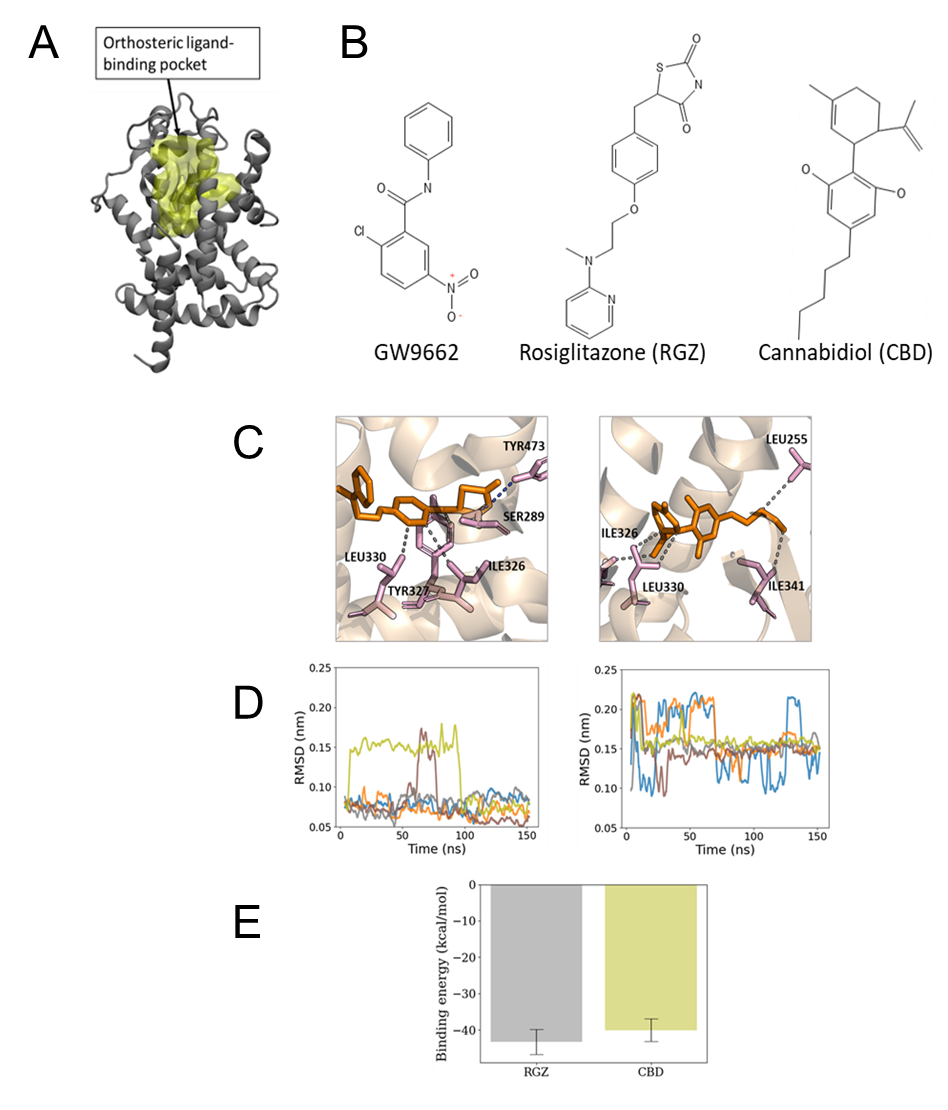
**

Supplemental Figure 7. (A) The structural location of PPAR-γ orthosteric ligand-binding pocket was identified by docking studies for analysis of the interaction with ligands. (B) The chemical structures of GW9662 (GW), rosiglitazone (RGZ), and cannabidiol (CBD) are shown in their 2d representative projection. (C) Binding poses for structures at the last frame from a representative replica over 150 ns of MD simulations for PPAR-γ-RGZ (left) and PPAR-γ-CBD (right), indicating the interactions with critical residue that contributed significantly to the binding of ligands in the orthosteric site. (D) Root-mean-square deviations (RMSD) of RGZ (left) and CBD (right) of five independent replicas during 150 ns of MD simulation. (E) The average of the relative binding free energies (in kcal/mol) of RGZ and CBD bounded to PPAR-γ were computed from the last 50 ns of five MD replicas. Data are presented as mean ± SEM.

**REFERENCES**

1. Avila-Barrientos LP, Cofas-Vargas LF, Agüero-Chapin G et al. Computational Design of Inhibitors Targeting the Catalytic β Subunit of *Escherichia coli* F O F 1 -ATP Synthase. Antibiotics (Basel) 2022;11.

2. Jang JY, Bae H, Lee YJ et al. Structural Basis for the Enhanced Anti-Diabetic Efficacy of Lobeglitazone on PPARγ. Sci Rep 2018;8:31.

3. Sali A, Blundell TL. Comparative protein modelling by satisfaction of spatial restraints. J Mol Biol 1993;234:779-815.

4. Eastman P, Swails J, Chodera JD et al. OpenMM 7: Rapid development of high performance algorithms for molecular dynamics. PLoS Comput Biol 2017;13:e1005659.

5. Darden T, York D, Pedersen L. Particle mesh Ewald: An N⋅log(N) method for Ewald sums in large systems. The Journal of Chemical Physics 1993;98:10089-10092.

6. Miyamoto S, Kollman PA. Settle: An analytical version of the SHAKE and RATTLE algorithm for rigid water models. Journal of Computational Chemistry 1992;13:952-962.

7. Wang J, Wolf RM, Caldwell JW, Kollman PA, Case DA. Development and testing of a general amber force field. Journal of Computational Chemistry 2004;25:1157-1174.

8. Tian C, Kasavajhala K, Belfon KAA et al. ff19SB: Amino-Acid-Specific Protein Backbone Parameters Trained against Quantum Mechanics Energy Surfaces in Solution. J Chem Theory Comput 2020;16:528-552.

9. Case DA, Aktulga HM, Belfon K et al. Amber 2022. University of California, San Francisco, 2022.

10. Miller BR, McGee TD, Swails JM, Homeyer N, Gohlke H, Roitberg AE. MMPBSA.py: An Efficient Program for End-State Free Energy Calculations. J Chem Theory Comput 2012;8:3314-21.
